# Wine‐Processed *Cornus officinalis* Ameliorates Osteoarthritis via Modulating M1/M2 Macrophage Polarization

**DOI:** 10.1111/jcmm.71113

**Published:** 2026-03-27

**Authors:** Yongsheng Fu, Minghua Zhao, Xudong Huang, Yingchao Ren, Weiguo Wang

**Affiliations:** ^1^ First Clinical Medical College Shandong University of Traditional Chinese Medicine Jinan China; ^2^ University Town Hospital Affiliated to Shandong University of Traditional Chinese Medicine Jinan China; ^3^ Affiliated Hospital of Shandong University of Traditional Chinese Medicine Jinan China

**Keywords:** apoptosis, CD206, CD86, inflammation, macrophage polarization, osteoarthritis, wine‐processed *Cornus officinalis*

## Abstract

*Cornus officinalis* (CO) is a traditional herbal medicine renowned in Traditional Chinese Medicine (TCM) for its properties of tonifying the liver and kidney and replenishing vital essence. Meanwhile, wine‐processed CO (pCO) exhibits superior pharmacological effects, including anti‐inflammatory, antioxidant and anti‐fibrotic activities. However, the immunomodulatory mechanism of pCO in osteoarthritis (OA) remains unclear. OA models were established in Sprague–Dawley rats via anterior cruciate ligament transection (ACLT). Network pharmacology and molecular docking were used to predict potential targets of pCO against OA, which were validated through behavioural tests, histomorphological staining and immunohistochemistry. HPLC‐Q‐Orbitrap‐MS analysis identified key differential compounds between raw and wine‐processed CO. The immunomodulatory effects of pCO were further confirmed by ELISA, immunofluorescence staining and RT‐qPCR. pCO ameliorated joint pain and cartilage damage in OA rats by reducing pro‐inflammatory factors (IL‐1β, COX‐2, IL‐12) and promoting anti‐inflammatory factors (IL‐10, TGF‐β1) in serum and synovial fluid. Network pharmacology combined with in vivo experiments revealed that pCO attenuated chondrocyte degeneration and apoptosis. Mechanistically, pCO modulated macrophage polarization by suppressing the M1 phenotype (CD86, iNOS) while promoting the M2 phenotype (CD206, TGF‐β1, Arg‐1), which revealed the key mechanism underlying its therapeutic effects against OA. pCO improved joint function and attenuated cartilage degeneration and synovial lesions, which were associated with the promotion of articular cartilage protection via the modulation of M1/M2 macrophage polarization.

**Abbreviations**
ACLT: aruciate ligament transection
BP: biological process
CC: closeness centrality
CCs: cellular components
DC: degree centrality
DL: drug‐likeness
ELISA: enzyme‐linked immunosorbent assay
GO: Gene Ontology
H&E: haematoxylin and eosin
IL: interleukin
KEGG: Kyoto Encyclopedia of Genes and Genomes
MF: molecular function
OA: osteoarthritis
OARSI: Osteoarthritis Research Society International
OB: oral bioavailability
pCO: wine‐processed *Cornus officinalis*

PPI: protein–protein interaction
TCMSP: Traditional Chinese Medicine Systems Pharmacology Database
TNF: tumour necrosis factor
TUNEL: terminal deoxynucleotidyl transferase dUTP nick end labeling

## 1 Introduction

Osteoarthritis (OA) is a chronic, progressive and degenerative disease characterized by articular cartilage damage, synovitis and structural changes in the subchondral bone [1]. The symptoms of OA include pain, stiffness and reduced range of motion, which can lead to functional limitations and severely impact patients' quality of life [2]. Among the pathogenic factors of OA, age and obesity are significant risk factors for disease progression. Currently, approximately 302 million people worldwide are affected by OA [3]. Noteworthy is the accumulating body of evidence underscoring that the release of inflammatory factors and degrading proteases into the synovial fluid within the joint, which leads to synovial fibrosis and cartilage damage, thereby disrupting the physiological metabolic crosstalk and functional homeostasis of chondrocytes [4]. Despite the high prevalence of OA, there are no long‐term effective disease‐modifying OA drugs (DMOADs) that can thoroughly alleviate pain and repair damaged cartilage [5]. Numerous anti‐OA drugs have been explored, including nonsteroidal anti‐inflammatory drugs (NSAIDs), which can protect and repair the cartilage layer. However, the long‐term application and potential liver and kidney toxicity limit their therapeutic efficacy in OA [6]. Therefore, this situation underscores the imperative need for rigorous examination and validation of therapeutic agents to develop safer and more effective treatment strategies for OA.

In recent years, natural herbs have gained wide attention for their extensive pharmacological components and diverse chemical properties, holding substantial promise for applications in daily life and medical development [7]. Although raw natural herbs possess broad anti‐inflammatory and antioxidant properties, such as resveratrol and curcumin, the dose safety and bioavailability present certain limitations in clinical OA applications [8, 9]. *Cornus officinalis* (CO), the dried raw fruit of the Cornus genus, exhibits anti‐inflammatory, antioxidant, antidiabetic and neuroprotective pharmacological effects [10]. A previous study demonstrated that natural CO reduced IL‐6 and TNF‐α levels to inhibit pro‐inflammatory factor production and impeded osteoclast differentiation, thereby alleviating femoral head osteonecrosis [11]. Xu et al. revelled that CO suppressed A375 tumour cell proliferation and melanoma angiogenesis by reducing serum levels of IL‐6, IL‐17A and IFN‐γ [12]. However, accumulating evidence suggested that wine‐processed herbs induced subtle changes in phytochemical composition, including increased abundance of bioactive metabolites and reduced toxic side effects, demonstrating enhanced potential against musculoskeletal diseases and fibrotic pathological progression [13, 14, 15, 16, 17]. Research has demonstrated that the effects of wine‐processed CO (pCO) are superior to those of raw CO, including dehydration‐induced changes in glycosidic bonds of iridoid glycosides. Notably, pCO significantly ameliorated liver fibrosis without hepatotoxicity, as evidenced by the downregulation of IL‐1β, F4/80, NLRP3 and caspase‐1 expression [18]. pCO also plays a significant role in ameliorating renal fibrosis and tissue damage, modulating Wnt/β‐catenin pathway expression and gut microbiota to inhibit inflammatory factor release with a favourable safety profile [19]. Liu et al. revealed increased abundance of iridoid compounds in pCO, with the 36‐fold enhancement in anti‐glucose effects, providing potential for biotransformation as an antidiabetic dietary supplement with improved efficacy and reduced toxicity [20]. Morroniside and loganin, as the primary iridoid compounds in raw CO [21], have been validated in multiple studies to reduce NLRP3 inflammasome activity through modulation of NF‐κB inflammatory pathway, inhibiting PGE2, MMP3 and MMP13 expression to alleviate OA cartilage inflammation and matrix degeneration [22, 23]. Although raw CO has been confirmed to possess medicinal potential in inhibiting pro‐inflammatory cytokine release and mitigating OA progression, the anti‐OA effects and anti‐inflammatory activity of pCO remain to be elucidated. Importantly, current research lacks investigation into the regulatory mechanism of pCO on M1/M2 macrophages in OA.

**FIGURE 1:**
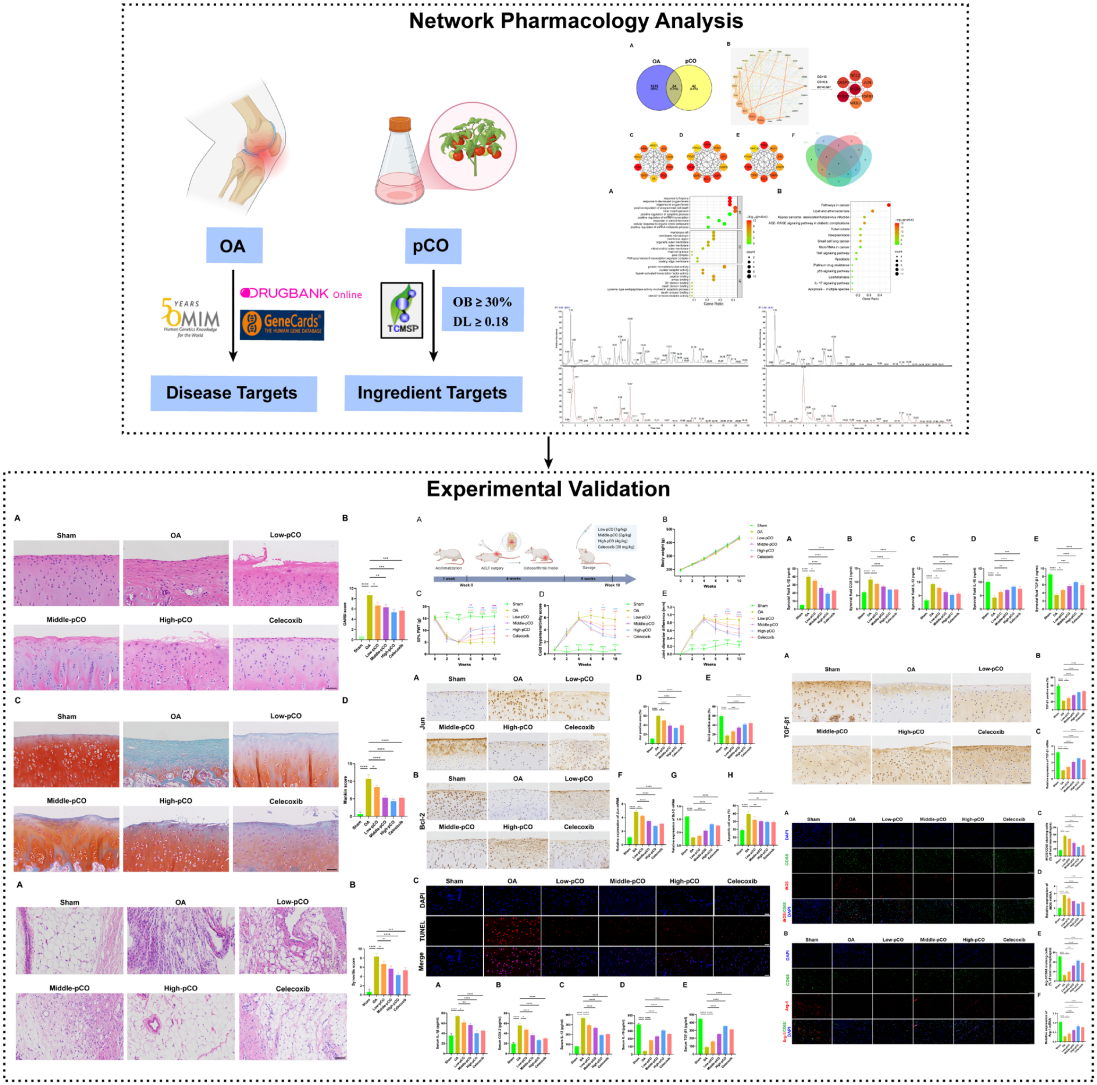
Flowchart of the current study strategy.

Network pharmacology, an emerging bioinformatics approach integrating phytochemical compounds with disease targets, holds significant promise for exploring and elucidating the targets and biological pathway mechanisms of drugs in the management of OA [24]. In this study, we simultaneously employed High‐Performance Liquid Chromatography coupled with Quadrupole Orbitrap Mass Spectrometry (HPLC‐Q‐Orbitrap‐MS) analysis to identify key differential components between CO and pCO involved in OA intervention. In vivo experiments using a rat OA model revealed the potential therapeutic effects of pCO on OA and further investigated the regulatory mechanism of pCO on M1/M2 macrophage polarization. These findings may provide theoretical value for preliminary experimental exploration and clinical application of pCO. Figure 1 illustrates the research strategy employed in this study to investigate the effects of pCO against OA.

## 2 Materials and Methods

### 2.1 Reagents and Materials

Bone joint decalcification fluid (#AR1071) and tissue fixation fluid (#AR1069) were purchased from Bosterbio Biological Engineering Co. Ltd. (Wuhan, China). The BCA protein concentration assay kit (#P0010) was purchased from Shanghai Beyotime Biotechnology Co. Ltd. (Shanghai, China). Haematoxylin and eosin staining kit (#G1120), Safranin O‐Fast Green cartilage staining kit (#G1371) and TUNEL apoptosis assay kit (#T2195) were purchased from Beijing Solarbio Technology Co. Ltd. (Beijing, China). Serum and synovial fluid ELISA kits for IL‐1β (#JYM0419), COX‐2 (#JYM0885), IL‐12 (#JYM1006), IL‐10 (#JYM0651) and TGF‐β1 (#JYM0636) were purchased from Wuhan GeneBeautiful Biological Engineering Co. Ltd. (Wuhan, China). Aspartate aminotransferase (AST) kit (#CSB‐E13023r) and alanine aminotransferase (ALT) kit (#CSB‐E13024r) were purchased from Cusabio Biotech Co. Ltd. (Wuhan, China). Urea nitrogen (BUN) assay kit (#C013‐2‐1) and creatinine (CR) assay kit (#C011‐2‐1) were purchased from Nanjing Jiancheng Bioengineering Institute (Nanjing, China). Jun Antibody (#24909–1‐AP), CD86 antibody (#13395–1‐AP), CD206 antibody (#18704–1‐AP), iNOS antibody (#22226–1‐AP) and Arg‐1 antibody (#16001–1‐AP) were purchased from Proteintech Group Inc. (Wuhan, China). Bcl‐2 antibody (# SZ10‐03) and TGF‐β1 antibody (#GB11285‐100) were purchased from HuaAn Biotechnology Co. Ltd. (Hangzhou, China). The HRP‐conjugated goat anti‐rabbit IgG (H+L) secondary antibody (#AS014) was purchased from ABclonal Technology Co. Ltd. (Wuhan, China). The raw CO, pCO drugs and celecoxib capsules required for the experiment were provided by the Affiliated Hospital of Shandong University of Traditional Chinese Medicine.

### 2.2 Animals

Thirty‐six male Sprague–Dawley (SD) rats, weighing 180–220 g, were purchased from Jinan Peng Yue Laboratory Animal Co. Ltd. (Jinan, China). All animals were acclimatized for 1 week prior to the experiments under standard laboratory conditions with constant humidity (50% ± 10%) and temperature (24°C ± 2°C), and a 12‐h light/dark cycle.

### 2.3 Potentially Active pCO Compounds and Their Targets

TCMSP database (https://old.tcmsp‐e.com/tcmsp.php) was used to determine potential targets of pCO ingredients. The potential active components of pCO were selected by oral bioavailability (OB ≥ 30%) and drug‐likeness (DL ≥ 0.18), and the core targets were further identified from pCO by screening in the TCMSP database [25]. All targets of the potential active ingredients were converted to *Homo sapiens* gene symbols using the UniProt database [26]. A compound‐target network of pCO was constructed and visualized using Cytoscape.

### 2.4 Screening of OA‐Associated Disease Targets

OA‐related gene targets were identified by searching the GeneCards (https://www.genecards.org/), OMIM (https://www.omim.org/) and DrugBank (https://go.drugbank.com/) databases using “osteoarthritis” as a keyword. The retrieved OA‐associated genes were merged and duplicates were removed [27]. The Venny 2.1 platform (https://bioinfogp.cnb.csic.es/tools/venny/) was employed to visualize the overlapping targets between pCO and OA.

### 2.5 Protein–Protein Interaction (PPI) Network Analysis

Common therapeutic targets between pCO and OA were identified through systematic analysis using the STRING database (version 11.5), with the organism parameter set to *Homo sapiens* . Interaction networks were constructed using a medium confidence threshold (combined score ≥ 0.4) and exported in TSV format for subsequent analysis. Network data were imported into Cytoscape (version 3.7.1) for topological characterization using the Network Analyser tool. To identify potential hub targets associated with the therapeutic mechanisms of pCO anti‐OA, the CytoHubba plugin was employed to screen for hub targets using distinct topological algorithms, including degree centrality (DC), betweenness centrality (BC), closeness centrality (CC), Maximal Clique Centrality (MCC), Maximum Neighbourhood Component (MNC) and Edge Percolated Component (EPC). Targets identified by the intersection of all three screening methods were defined as core targets.

### 2.6 Gene Ontology (GO) and Kyoto Encyclopedia of Genes and Genomes (KEGG) Pathway Enrichment Construction

The results of GO and KEGG pathway enrichment analyses were visualized using the Metascape database (https://metascape.org/gp/index.html). The *p*‐value and minimum overlap in the results of enrichment analyses were set to 0.01 and 3, respectively. GO enrichment analyses were conducted across three categories: biological processes (BPs), cellular components (CCs) and molecular functions (MFs). The processed data were graphically displayed using bioinformatics visualization software (https://www.bioinformatics.com.cn/), with emphasis on the most significant GO terms and top‐ranked KEGG pathways through bubble diagram visualization for mechanistic interpretation [28]. A network composed of drug component‐target‐pathway‐disease (D‐C‐T‐P‐D) was constructed using Cytoscape 3.7.2, which established a systematic evaluation of node centrality measures and interaction patterns among network components.

### 2.7 Molecular Docking

The identified compounds were queried in the PubChem database to download their respective 2D structures. These structures were energy‐minimized using Chem3D software and saved in ‘mol2’ format, thereby obtaining the small molecule ligands associated with the core components. The 3D structures of proteins corresponding to the core target genes were retrieved from the Protein Data Bank (PDB) database (https://www.rcsb.org/). Using PyMOL software, water molecules and heterogeneous small molecules were removed from the proteins, and the resulting structures were saved in “PDBQT” format to generate the protein receptors for the core targets [29, 30]. Molecular docking of the small‐molecule ligands to the protein receptors was performed using the AutoDockTools (version 2.5.1) software. Subsequently, molecular docking was performed using AutoDockTools with a grid box centered on the active site of the protein. The grid box dimensions were adjusted to encompass both the original ligand and entire protein receptor structure. The ligand structures were parameterized to achieve low‐energy binding conformations, and output files were generated in the PDBQT format. PyMOL was employed to visualize the binding sites and identify key interaction residues between critical proteins and ligands. The highest‐scoring conformations were visualized using PyMOL software and Discovery Studio 2019.

### 2.8 HPLC‐Q‐Orbitrap‐MS Analysis

Frozen samples were thawed at room temperature and vortexed for 30 s to ensure thorough mixing. Subsequently, the samples were centrifuged at 13,000 rpm for 10 min at 4°C. An aliquot of 200 μL of the supernatant was transferred to a 1.5 mL centrifuge tube, and 1000 μL of methanol–water (4:1, v/v) extraction solvent was added, followed by vortex mixing for 10 min. The mixture was centrifuged again at 13,000 rpm for 10 min at 4°C. The resulting supernatant was filtered through a 0.22 μm organic membrane filter, and the filtrate was collected into an HPLC vial for subsequent analysis. Chromatographic separation was performed on a Welch AQC18 column (1.8 μm, 150 × 2.1 mm). The mobile phase consisted of water containing 0.1% formic acid (A) and methanol (B), with a flow rate of 300 μL/min and an injection volume of 5 μL. Gradient elution was programmed as follows: 0–5 min, 2% to 20% B; 5–10 min, increased to 50% B; 10–15 min, increased to 80% B; 15–20 min, increased to 95% B, and maintained until 27 min (Table S1). Mass spectrometric analysis was performed using an electrospray ionization (ESI) source in positive ion mode. The main parameters were set as follows: sheath gas flow rate, 40 Arb; auxiliary gas flow rate, 15 Arb; spray voltage, 3.2 kV; capillary temperature, 300°C. The full scan resolution was set at 70,000, and the MS/MS resolution at 17,500. High‐purity argon (purity ≥ 99.999%) was used as the collision gas, with a normalized collision energy (NCE) of 30%. The data acquisition time was 30 min.

### 2.9 Establishment and Treatment of the OA Model

Animal models of OA were established by anterior cruciate ligament transection (ACLT) of the right knee joint in SD rats [31]. Thirty‐six SD rats were randomly allocated to six experimental groups, with six rats in each group. The experimental groups included the sham group, OA group, low‐pCO group, middle‐pCO group, high‐pCO group and celecoxib group. Except for the sham group, all rats underwent ACLT surgery. The low, middle and high doses of the pCO therapeutic drugs were administered at a ratio of 1:2:4 (1, 2 and 4 g/kg pCO, respectively) [32, 33]. The dose for the high‐pCO rats was converted from the clinical human equivalent dose based on body surface area (6.3:1) [34]. OA rats in the celecoxib group were treated daily with celecoxib capsules (20 mg/kg). The specific oral dosage was determined using an animal dose conversion formula. Four weeks after modelling, SD rats in each group received pharmacological intervention by oral gavage once daily for 6 weeks. Rats in the sham and OA groups were gavaged with an equal volume of saline.

### 2.10 Animal Behaviour Testing

Following ACLT surgery, behavioural tests were conducted on rats in each group every 2 weeks, including assessment of mechanical pain threshold (Von–Frey Test), cold sensitivity test and joint swelling measurement. Mechanical allodynia was assessed using calibrated von Frey monofilaments applied to the center of the plantar surface of the right hind paw via the up‐down method. Following a stimulation period of 3–5 min, positive withdrawal responses were recorded. The 50% paw withdrawal threshold (PWT) was calculated to quantify mechanical sensitivity [35, 36, 37]. Cold sensitivity test: Acetone was applied to the centre of the plantar surface of the right hind paw, and behavioural changes in the rats were observed and recorded. Rat behaviours were scored on a scale of 0 to 4 [38]. Knee joint diameter measurement: The rat knee joints were extended to their maximum, and the diameter was measured using a digital calliper. Measurements were accurately recorded [39].

### 2.11 Histopathologic Staining and Analysis

After the intervention, the harvested rat knee tissues were fixed in a 4% paraformaldehyde solution for 2 days in preparation for subsequent staining procedures. Paraffin‐embedded sections (4 μm) of decalcified cartilage were deparaffinized in xylene, rehydrated through graded ethanol, and then stained with haematoxylin and eosin (H&E) and Safranin O/Fast Green (S‐O) staining. After air‐drying and sealing the sections with a neutral gel, the morphological structure of the cartilage in each group was observed under a light microscope. The severity of cartilage degeneration was critically evaluated using the Osteoarthritis Research Society International (OARSI) and Mankin scoring systems, with scores ranging from 0 to 24 and 0 to 14, respectively [40]. In addition, the knee synovium was isolated, and 4 μm paraffin sections were prepared. The sections were subjected to H&E staining to assess inflammatory cell infiltration in the joint tissue. The histopathological grading of synovitis was based on Krenn's Synovitis Scale (0–9) [41].

### 2.12 Immunohistochemistry Analysis

Cartilage and synovial tissues from the rats in each group underwent antigen retrieval following paraffin sectioning and dewaxing hydration. After membrane washing, the sections were treated with peroxidase blocking solution at room temperature for 20 min. Primary antibodies were then applied dropwise to cartilage and synovial tissue sections and incubated at 4°C overnight. Subsequently, the sections were incubated with the secondary antibodies at room temperature for 30 min. DAB staining was performed, followed by counterstaining with haematoxylin. The sections were then sealed with xylene. Finally, the sections were examined under a microscope, and images were captured for analysis.

### 2.13 Reverse Transcription‐Quantitative Real‐Time PCR (RT‐qPCR) Analysis

Cartilage and synovial tissues were collected separately from rat knee joints. Protease inhibitors and grinding beads were added to the EP tubes containing the different tissues, and the samples were homogenized using a low‐temperature grinding mill until a uniform consistency was achieved. After treatment with enzyme‐free water, isopropanol and 75% ethanol, the precipitate dissolved in enzyme‐free water was obtained as total RNA and the concentration of total RNA was measured using a nucleic acid protein assay. The RNA was reverse‐transcribed into first‐strand cDNA using a reverse transcription kit, and cDNA was amplified using PrimeScript RTregent reagent. PCR reaction system: 10 μL qPCR Premix, 0.4 μL each of forward and reverse primers, 1 μL of DNA template, 8.2 μL of Enzyme‐free water. The qPCR procedure was as follows: 45 cycles of pre‐denaturation at 95°C for 30 s, denaturation at 95°C for 20 s and annealing and extension at 60°C for 30 s. β‐actin was selected as the internal reference gene, and relative gene expression was calculated using the 2^−ΔΔCT^ method. The primer sequences are shown in Table 1.

**TABLE 1:** The primers for qPCR.

Genes	Forward (5′‐3′)	Reverse (5′‐3′)
Jun	GCACATCACCACTACACCGA	TATGCAGTTCAGCTAGGGCG
Bcl‐2	TGGCCTTCTTTGAGTTCGGT	GTTCCACAAAGGCATCCCAGC
TGF‐β1	TGACATGAACCGACCCTTCC	TGTGGAGCTGAAGCAGTAGT
CD86	TCTAAGCGCCATCTCCGTTC	ATGATCTGCGACTCCGACAC
CD206	CGAACACTATTTGGGCGCAG	CAAGCCCGTGTCCTTGATCT
iNOS	TGGCCACCTTGTTCAGCTAGG	GCCAAGGCAACACAGGATAC
Arg‐1	CTCCAAGCAAGTCCTTAGAG	AGGAGCTGTCATTAGGACATG
β‐Actin	CCATTCTATGAGGTGCACGC	TGCAGTGTCACGACGAGGTC

### 2.14 Terminal Deoxynucleotidyl Transferase dUTP Nick End Labeling (TUNEL) Analysis

TUNEL staining was performed on completely decalcified rat cartilage tissue to detect chondrocyte apoptosis in OA rats. The protocol involved dewaxing and rinsing the tissue with PBS. Cartilage tissue was dewaxed and dehydrated using proteinase K. After incubation at ambient temperature, 25 μL of TUNEL reaction reagent solution was added. After incubation for 1 h at 37°C in a wet box, haematoxylin restaining and xylene transparent sealing were performed. Haematoxylin was used for counterstaining, followed by dehydration and mounting. Apoptotic cells were identified using red staining. Finally, images were acquired and analysed to quantify the apoptotic regions and proportions.

### 2.15 Serum Analysis

Blood samples were rapidly centrifuged and stored at −80°C before use in triplicate for each sample. An ELISA kit was used to assess the level of inflammation in rat serum. The ELISA instrument was set at 450 nm for absorbance (OD) detection and analysis of IL‐1β, COX‐2, IL‐10, IL‐12 and TGF‐β1.

### 2.16 Synovial Fluid Analysis

Synovial fluid (100 μL per rat) was collected from each group and stored at −80°C until analysis. Joint synovial fluid samples were centrifuged at 3000*g* for 5 min at 4°C to obtain the supernatant. Levels of the biomarkers IL‐1β, COX‐2, IL‐10, IL‐12 and TGF‐β1 were measured using commercial ELISA kits according to the manufacturer's instructions.

### 2.17 Oral Toxicity Analysis

Serum levels of alanine aminotransferase (ALT), aspartate aminotransferase (AST), creatinine (Cr) and blood urea nitrogen (BUN) in each group of rats were measured using an automatic biochemical analyser. Liver, kidney and lung tissues were harvested from rats in each experimental group and subjected to H&E staining. Pathological changes in rat tissues were observed and analysed under a light microscope to verify the potential toxic effects of pCO in vivo.

### 2.18 Immunofluorescence Analysis

Paraffin‐embedded sections of synovial tissue were deparaffinized with xylene, rehydrated through a graded ethanol series and washed with PBS. Antigen retrieval was performed by incubating the sections in citrate buffer. The sections were then blocked with 5% bovine serum albumin (BSA) at room temperature for 30 min to prevent non‐specific binding. Subsequently, the sections were incubated with primary antibodies overnight at 4°C. After washing, the sections were incubated with species‐appropriate secondary antibodies at room temperature for 2 h. Nuclei were counterstained, and the sections were washed, dried and visualized under a fluorescence microscope. Co‐localization analysis was performed using Image J software.

### 2.19 Statistical Analysis

The experimental data were analysed and graphically represented using GraphPad Prism 9.5.1 software, the results are expressed as mean ± standard deviation (SD). Statistical comparisons between groups were performed using one‐way analysis of variance (ANOVA), supplemented by Tukey's multiple comparison test for post hoc analysis, and the difference was statistically significant when *p* < 0.05.

## 3 Results

### 3.1 Screening of pCO Biological Components and Overlapping Targets

**FIGURE 2:**
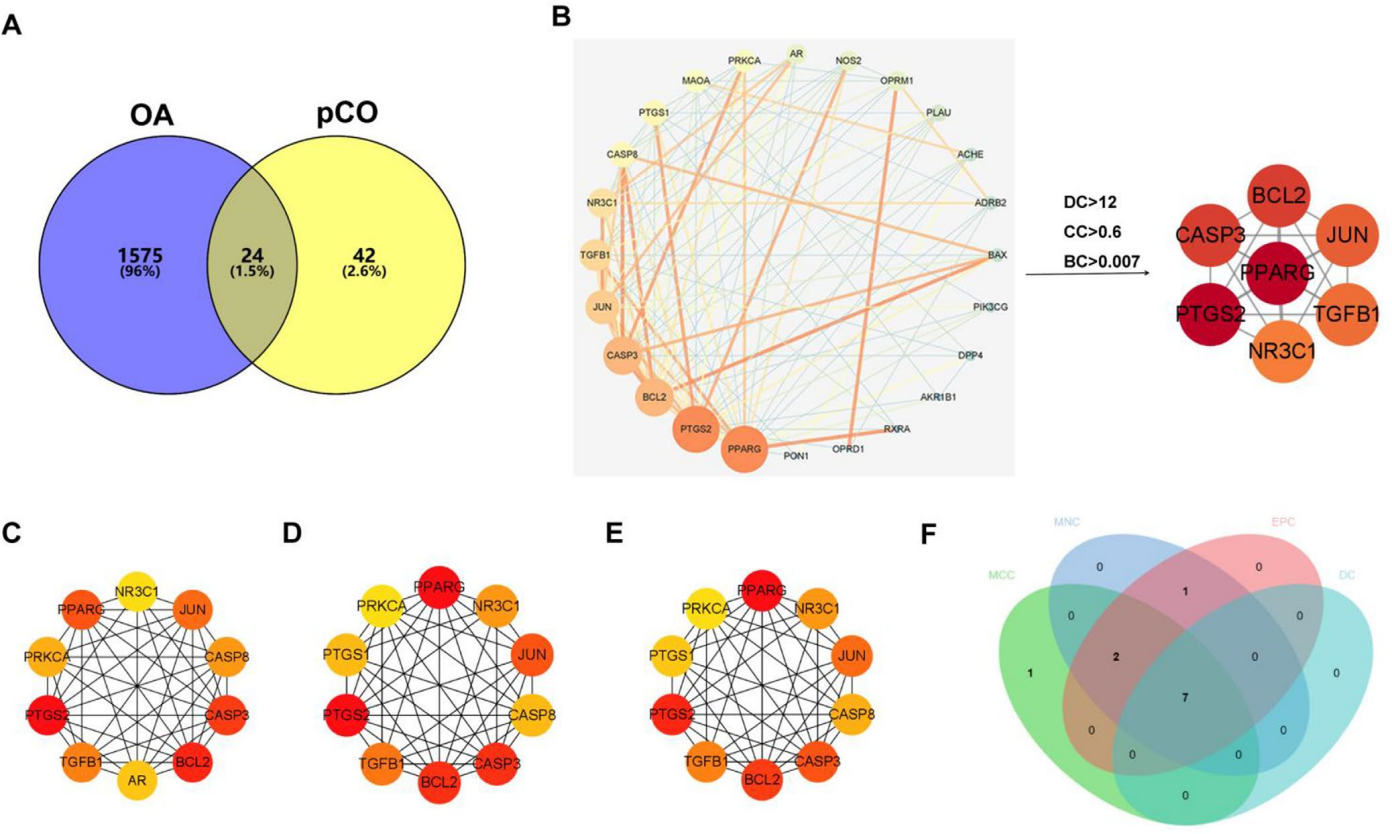
(A) Venn diagram illustrating the overlapping targets between pCO and OA. (B) Screened key targets from the PPI network of pCO against OA by DC, BC and CC values. (C) Core targets screened by MCC value in the PPI network. (D) Core targets screened by MNC value in the PPI network. (E) Core targets screened by EPC value in the PPI network. (F) The 7 core targets obtained through topological analysis, MCC, MNC and EPC methods in the PPI network.

The identified potential natural components and the corresponding targets are involved in the pharmacological mechanism of pCO against OA. Using the TCMSP database, 14 potential components were identified after removing components without known targets (Table S2). Subsequently, 66 pCO targets were determined after eliminating duplicates (Table 2). Using disease databases revealed 1599 potential OA‐related targets (Table S3). Analysis of the overlap between pCO and OA‐related targets identified 24 potential core targets (Figure 2A).

**TABLE 2:** Basic information on the 14 potential active components of pCO.

NO.	Molecule ID	Component	Formula	OB (%)	DL
pCO1	MOL001494	Mandenol	C_20_H_36_O_2_	42.00	0.19
pCO2	MOL001495	Ethyl linolenate	C_20_H_34_O_2_	46.10	0.20
pCO3	MOL001771	Poriferast‐5‐en‐3Beta‐ol	C_29_H_50_O	36.91	0.75
pCO4	MOL002879	Diop	C_24_H_38_O_4_	43.59	0.39
pCO5	MOL002883	Ethyl oleate	C_20_H_38_O_2_	32.40	0.19
pCO6	MOL003137	Leucanthoside	C_22_H_22_O_11_	32.12	0.78
pCO7	MOL000358	Beta‐sitosterol	C_29_H_50_O	36.91	0.75
pCO8	MOL000359	Sitosterol	C_29_H_50_O	36.91	0.75
pCO9	MOL000449	Stigmasterol	C_29_H_48_O	43.83	0.76
pCO10	MOL005481	2,6,10,14,18‐Pentamethylicosa‐2,6,10,14,18‐pentaene	C_25_H_42_	33.40	0.24
pCO11	MOL005503	Cornudentanone	C_22_H_34_O_5_	39.66	0.33
pCO12	MOL005530	Hydroxygenkwanin	C_16_H_12_O_6_	36.47	0.27
pCO13	MOL005531	Telocinobufagin	C_24_H_34_O_5_	69.99	0.79
pCO14	MOL008457	Tetrahydroalstonine	C_21_H_24_N_2_O_3_	32.42	0.81

### 3.2 Construction and Analysis of PPI Network

PPI network analysis revealed interactions between 24 potentially essential targets. A PPI network was constructed with 24 nodes and 111 edges representing potential therapeutic targets for pCO and OA. Topological analysis identified core targets based on DC, BC and CC, identified seven core targets with significant network topological features (Figure 2B) (Table S4). In the network visualization, node coloration followed a gradient spectrum from light to dark, corresponding to ascending degree values that reflected the target importance (Figure 2C–E). Furthermore, the CytoHubba plugin was used to screen the top 10 crucial targets through MCC, MNC and EPC (Figure 2F). Seven core targets (PPARG, PTGS2, BCL2, CASP3, JUN, TGFβ1 and NR3C1) were identified as the intersecting targets of MCC, MNC, EPC and topological network analysis, and were associated with the anti‐OA effects of pCO.

### 3.3 GO and KEGG Enrichment Analysis

**FIGURE 3:**
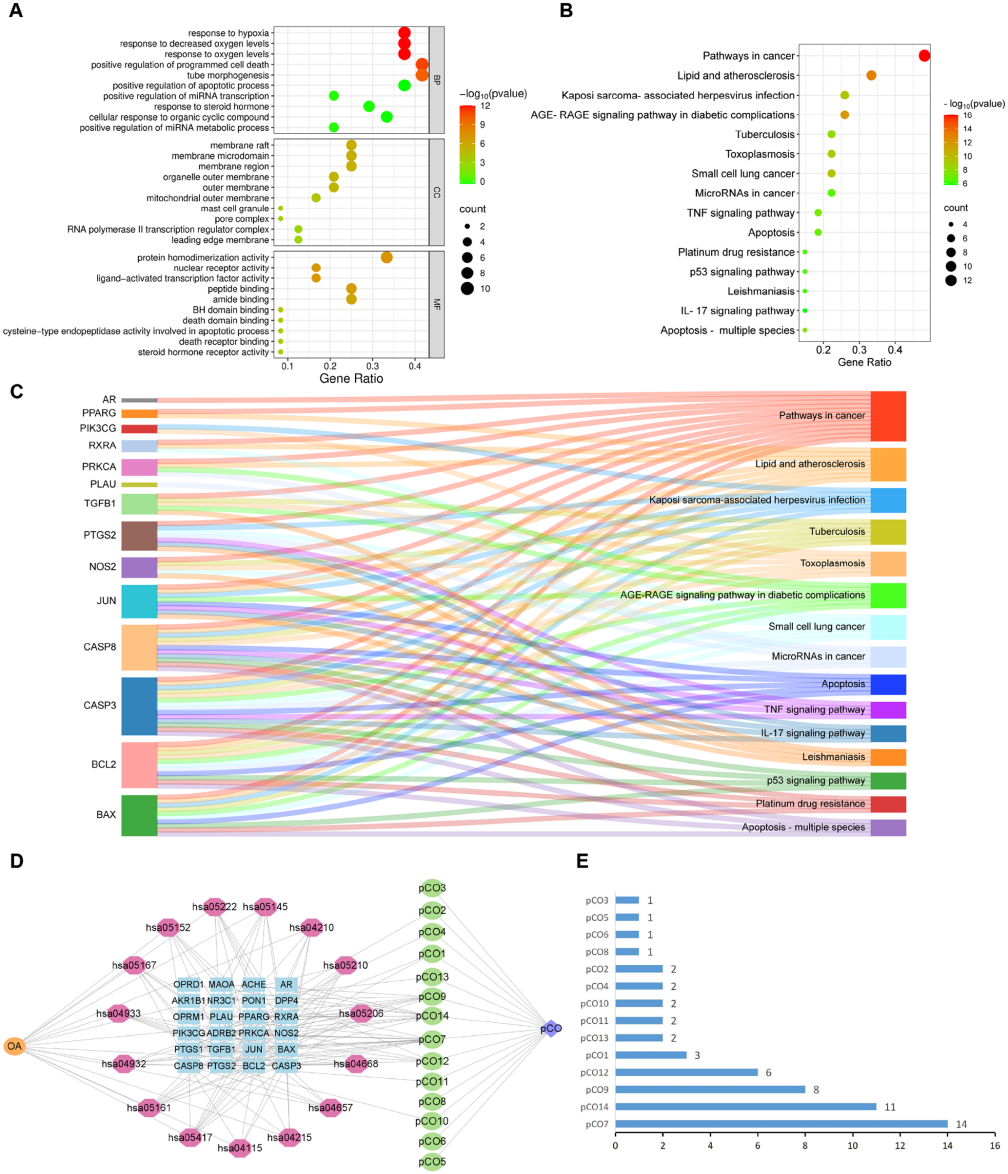
(A) Bubble plots of BP enrichment analysis of key targets of pCO for OA. (B) Bubble plot depicting the results of KEGG signalling pathway enrichment analysis. (C) Sankey diagram illustrating the KEGG pathway enrichment analysis of pCO targets in OA. (D) D‐C‐T‐P‐D network of pCO and OA targets. The orange circle represents the disease, pink diamonds represent pathways, blue squares represent OA core targets of pCO, green circles represent pCO compounds, and the purple arrow represents pCO. (E) Bar chart showing the degree values of connected pCO components.

GO enrichment analysis was performed using Metascape to investigate the biological functions and pathways associated with the anti‐OA effects of pCO. Based on the results, the top 10 enriched GO terms for BPs, CCs and MFs were selected (Figure 3A). The BP category was predominantly enriched in developmental processes such as response to hypoxia, response to decreased oxygen levels, and response to oxygen levels. CCs terms were significantly associated with membrane rafts, membrane microdomains and membrane regions. The MF annotations were mainly characterized by protein homodimerization activity, nuclear receptor activity and ligand‐activated transcription factor activity.

KEGG pathway enrichment analysis identified the top 15 pathways associated with the anti‐OA effects of pCO. These included pathways in cancer, IL‐17 signalling pathway, and apoptosis (Figure 3B). A Sankey diagram was used to visualize the connections between the enriched pathways and their corresponding targets (Figure 3C). Key signalling pathways associated with apoptosis and inflammatory mechanisms were implicated in the protective effects of pCO against OA progression. Cytoscape 3.7.2 was used to construct the network between the pCO components and disease targets (Figure 3D) (Table 3). Bar plots were generated to visualize the interaction degree values between the 14 compounds and their corresponding targets (Table S5), as well as the top 15 enriched pathways (Figure 3E).

**TABLE 3:** KEGG enrichment results of the top 15 enrichment pathways.

Number	KEGG pathways	Count	Genes	*p*
hsa05200	Pathways in cancer	12	AR, BAX, BCL2, CASP3, CASP8, JUN, NOS2, PPARG, PRKCA, PTGS2, RXRA, TGFB1	1E‐15
hsa05417	Lipid and atherosclerosis	8	BAX, BCL2, CASP3, CASP8, JUN, PPARG, PRKCA, RXRA	1E‐11
hsa05222	Small cell lung cancer	6	BAX, BCL2, CASP3, NOS2, PTGS2, RXRA	1E‐10
hsa04933	AGE‐RAGE signalling pathway in diabetic complications	6	BAX, BCL2, CASP3, JUN, PRKCA, TGFB1	1.58489E‐10
hsa05145	Toxoplasmosis	6	BCL2, CASP3, CASP8, NOS2, PIK3CG, TGFB1	2.51189E‐10
hsa05152	Tuberculosis	6	BAX, BCL2, CASP3, CASP8, NOS2, TGFB1	5.01187E‐09
hsa05167	Kaposi sarcoma‐associated herpesvirus infection	6	BAX, CASP3, CASP8, JUN, PIK3CG, PTGS2	7.94328E‐09
hsa04215	Apoptosis—multiple species	4	BAX, BCL2, CASP3, CASP8	0.00000001
hsa04210	Apoptosis	5	BAX, BCL2, CASP3, CASP8, JUN	6.30957E‐08
hsa01524	Platinum drug resistance	4	BAX, BCL2, CASP3, CASP8	3.16228E‐07
hsa04115	p53 signalling pathway	4	BAX, BCL2, CASP3, CASP8	3.16228E‐07
hsa05210	Leishmaniasis	4	JUN, NOS2, PTGS2, TGFB1	3.98107E‐07
hsa04657	IL‐17 signalling pathway	4	CASP3, CASP8, JUN, PTGS2	7.94328E‐07
hsa04668	TNF signalling pathway	4	CASP3, CASP8, JUN, PTGS2	1.99526E‐06
hsa05206	MicroRNAs in cancer	5	BCL2, CASP3, PLAU, PRKCA, PTGS2	3.98107E‐06

### 3.4 Molecular Docking

**FIGURE 4:**
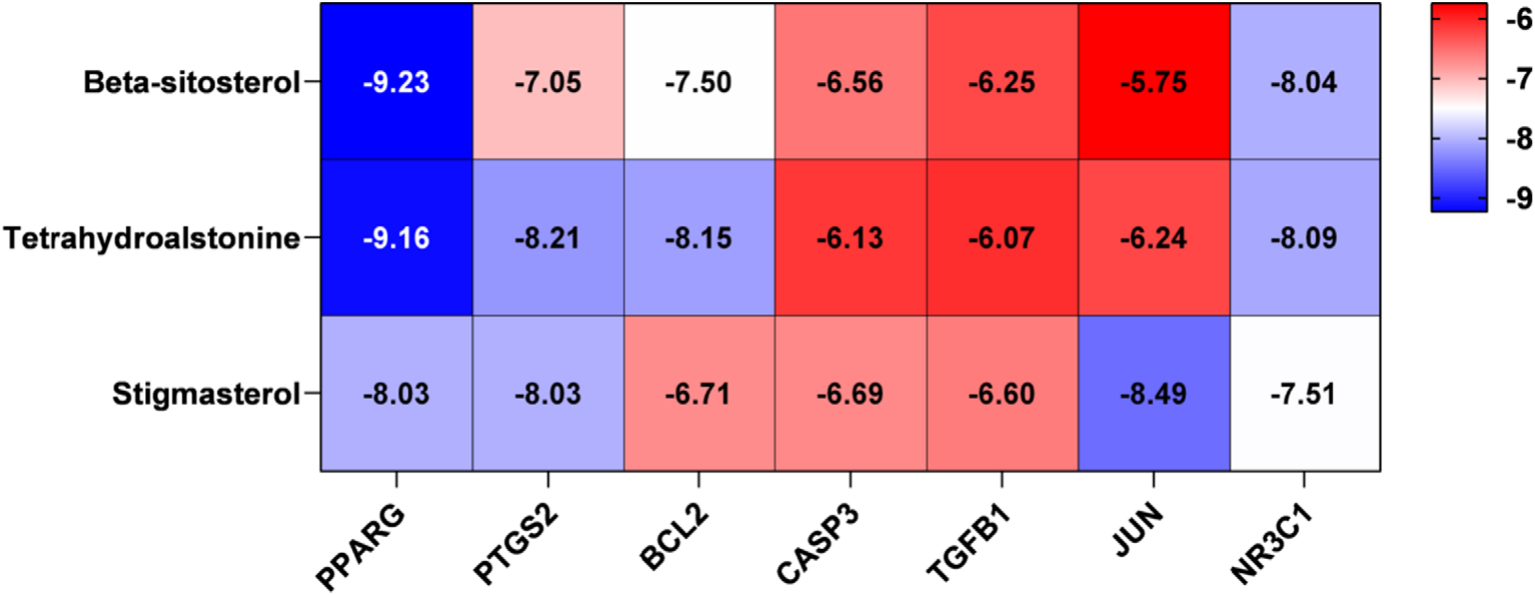
Molecular docking score heatmap. The binding energy (kcal/mol) scores of pCO with its predicted target proteins.

**FIGURE 5:**
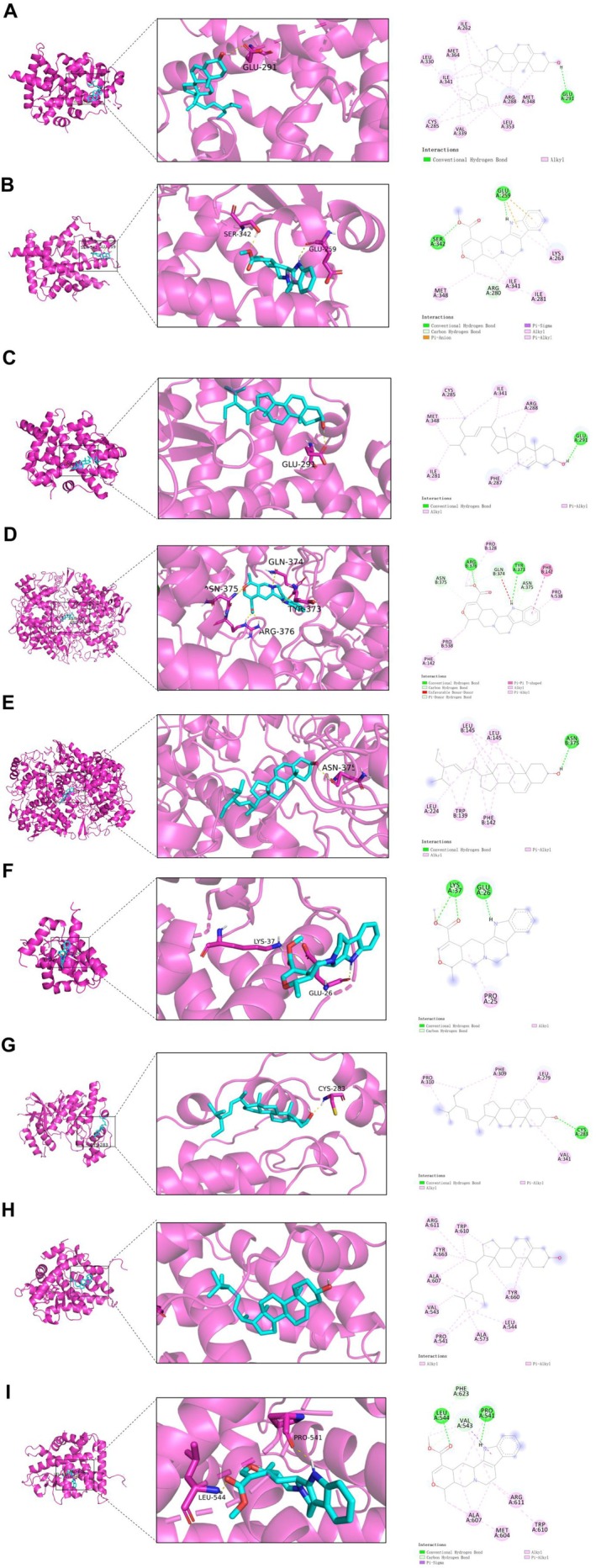
Molecular docking interactions between pCO components and core targets. (A‐C) Binding modes of Beta‐sitosterol, Tetrahydroalstonine and Stigmasterol with PPARG. (D‐E) Interactions of Tetrahydroalstonine and Stigmasterol with PTGS2. (F) Tetrahydroalstonine binding to Bcl‐2. (G) Stigmasterol binding to Jun. (H‐I) Beta‐sitosterol and Tetrahydroalstonine interactions with NR3C1.

Based on the network topology analysis, the top three compounds were selected for molecular docking validation. Figure 4 displayed the binding energies between the top three components and seven key targets. It is widely recognized that lower docking binding energies correspond to more stable conformations, with a binding energy lower than −5 kcal/mol, indicating favourable binding affinity [42]. Therefore, molecular complexes with binding energies below −8 kcal/mol were selected for docking analysis to elucidate their binding capabilities. The results revealed that PPARG exhibited the most favourable binding affinity for Beta‐sitosterol at −9.23 kcal/mol. The docking scores of PPARG with tetrahydroalstonine and stigmasterol were −9.16 kcal/mol and −8.03 kcal/mol, respectively. PTGS2 formed hydrogen bonds with tetrahydroalstonine at residue GLU259 and SER342, demonstrating a binding affinity of −8.21 kcal/mol, while its binding energy with stigmasterol reached −8.03 kcal/mol. The binding energies of Bcl‐2 with tetrahydroalstonine and JUN with stigmasterol were −8.15 kcal/mol and −8.49 kcal/mol, respectively. NR3C1 showed binding energies of −8.04 kcal/mol and −8.09 kcal/mol with Beta‐sitosterol and tetrahydroalstonine, respectively (Figure 5). These results indicated that pCO components interact with protein receptors through strong hydrogen bonds and multiple residue interactions involving GLU and SER. Although molecular docking analysis demonstrated the binding affinities of three components to the seven identified protein targets, the key active constituents responsible for the therapeutic effects of pCO against OA warrant further investigation.

### 3.5 Screening of Chemical Components in CO and pCO by HPLC‐Q‐Orbitrap‐MS

**FIGURE 6:**
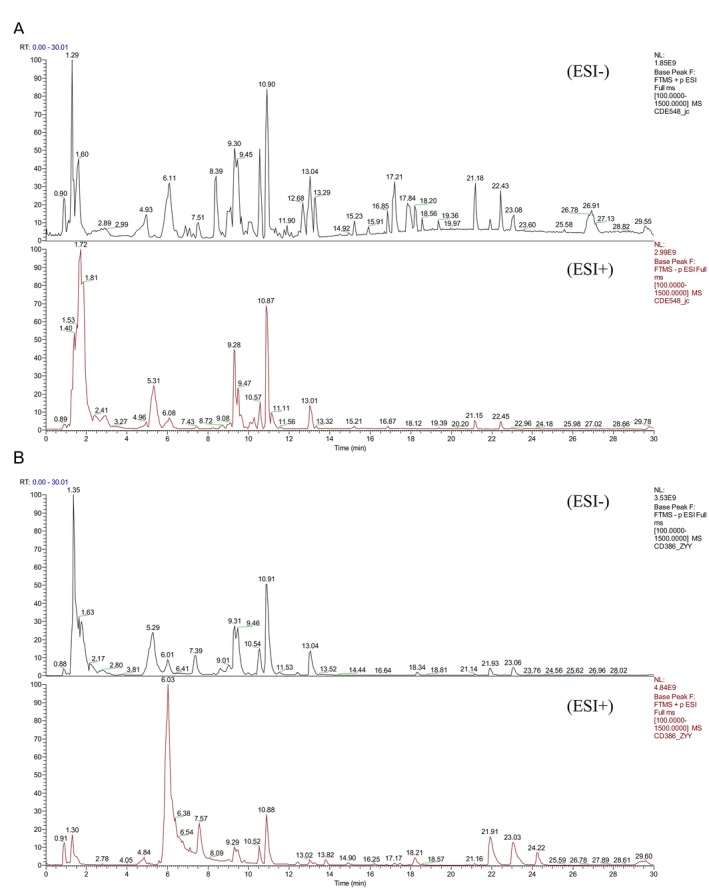
Identification of compounds in CO and pCO using HPLC‐Q‐Orbitrap‐MS. (A) Total ion chromatograms (TIC) of CO in positive and negative ion modes. (B) TIC of pCO in negative ion mode.

In this study, high‐resolution mass spectrometry data of CO and pCO obtained under both positive and negative ion modes were compared based on HPLC‐Q‐Orbitrap‐MS analysis. Representative total ion chromatograms illustrating compound profiling are shown in Figure 6. The identification of compounds in CO and pCO was performed through comparison with standard mass spectral databases, validation by existing literature and differential characterization analysis. A total of 42 key components were identified and screened.

**FIGURE 7:**
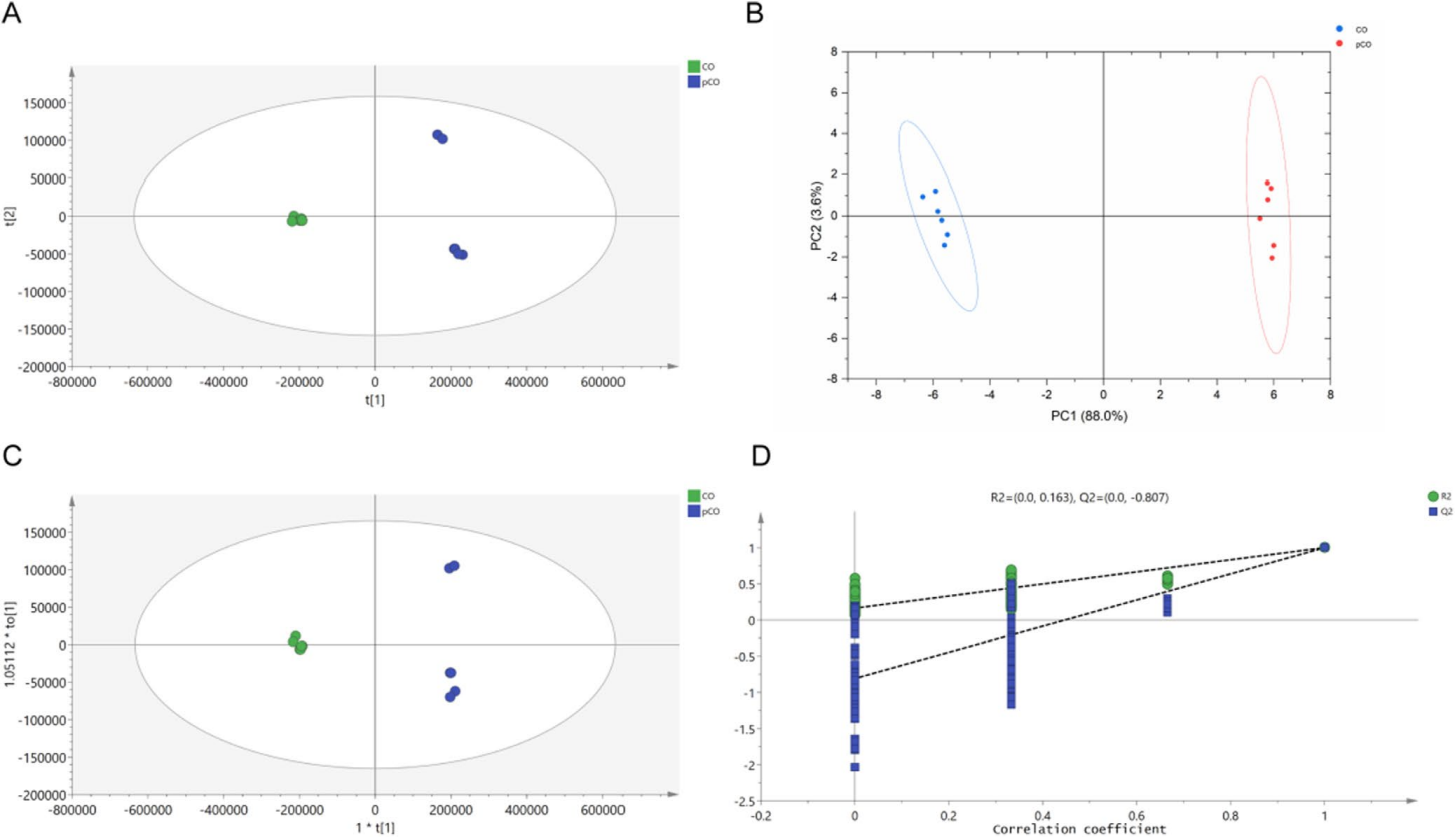
Analysis of metabolites of CO and pCO. (A) PCA‐X score (*R* ^2^ *X* = 0.962, *Q* ^2^ = 0.894). (B) PCA score plot (PC1 = 88%, PC2 = 3.6%). (C) OPLS‐DA score (*R* ^2^ *X* = 0.961, *R* ^2^ *Y* = 0.999 and *Q* ^2^ = 0.997). (D) Permutation test plot (200 permutations).

To identify significant compositional differences between the two samples, multivariate statistical analysis was performed using SIMCA 14.1 software. Principal component analysis (PCA‐X) was employed to visualize the metabolic differences between raw and processed CO samples (Figure 7A). The PCA score plot revealed a clear separation between the two groups, indicating substantial compositional changes induced by processing, with robust model parameters (*R* ^2^ *X* = 0.962, *Q* ^2^ = 0.894). Figure 7B further demonstrates the distinct separation between the two samples, validating the differences and reliability of their metabolic profiles (PC1 = 88%, PC2 = 3.6%). Orthogonal partial least squares discriminant analysis (OPLS‐DA) score plots exhibited significant inter‐group separation, consistent with the PCA results (*R* ^2^ *X* = 0.961, *R* ^2^ *Y* = 0.999, *Q* ^2^ = 0.997), enabling further identification of differential metabolites (Figure 7C). Permutation testing (200 iterations) yielded a *Q* ^2^ regression line with an intercept below zero (Figure 7D), confirming the robustness of the model without overfitting. Following comparison of mass spectrometry data, fragmentation patterns and literature validation, the detailed characteristics of 42 compounds are presented in Table 4. Taking loganin as an example, its molecular formula was calculated as C_17_H_26_O_10_, with an observed mass of 390.15316 Da (theoretical mass: 390.15260 Da), ion type [M‐H]^−^ and a key fragment at 227.09224 Da [43]. Comparison with published data confirmed the identification of this compound as loganin. Using a variable importance in projection (VIP) score > 1.0 as the screening criterion, 11 components were identified as significantly altered following processing. The present results also revealed trends in the chemical profiles of pCO compared to raw CO, including compounds such as luteolin, gallic acid and quercetin. Mechanistically, luteolin has been reported to inhibit CD74 expression, disrupting the CEBPB‐p65 complex assembly and thereby suppressing macrophage inflammatory factor activation in OA synovitis [44]. Quercetin modulated the cGAS/STING pathway, attenuating inflammatory factor synthesis and alleviating joint pain hypersensitivity [45]. Gallic acid scavenged reactive oxygen species in the joint and mitigated cartilage matrix damage caused by macrophage‐mediated inflammatory imbalance [46]. Collectively, these findings revealed the significant role of pCO in suppressing inflammation and modulating macrophage polarization.

**TABLE 4:** Chemical components identified by HPLC‐Q‐Orbitrap‐MS.

No.	Retention (min)	Molecular formula	m/z (theoretical)	Ion type	m/z (actual)	Error (ppm)	Name	MS/MS ions (m/z)	Intensity trend
1	1.39	C_5_H_11_NO_2_	117.07898	[M+H]+1	117.07925	2.35	Betaine	118.08655, 74.06060, 59.07383	↑
2	1.59	C_4_H_6_O_6_	150.01644	[M‐H]‐1	150.01538	−6.04	Tartaric acid	149.00826, 130.99759, 103.00243, 87.0745, 72.99176, 59.01249	↑
3	1.61	C_6_H_8_O_7_	192.02710	[M‐H]‐1	192.02658	−2.21	Citric acid	191.01917, 129.01820, 111.00755, 87.0745, 85.02818	↓
4	1.71	C_9_H_16_O_4_	188.10486	[M‐H]‐1	188.10462	−1.28	Azelaic acid	187.09712, 125.09610, 97.06465	↑
5	1.82	C_15_H_12_O_5_	272.06847	[M‐H]‐1	272.06886	1.44	Naringenin	273.07614, 171.02899, 153, 01831, 147.04414, 119.04946	↑
6	2.93	C_9_H_8_O_4_	180.04226	[M‐H]‐1	180.04163	−3.52	Caffeic acid	179.03435, 135.04407, 59.01249	↓
7	5.32	C_7_H_6_O_5_	170.02152	[M‐H]‐1	170.02065	−5.16	Gallic acid	169.01337, 125.02328, 97.02824, 81.03323, 69.03324	↑
8	6.13	C_15_H_10_O_6_	286.04774	[M‐H]‐1	286.04815	1.44	Luteolin	285.04095, 151.00266, 109.02834	↑
9	7.43	C_17_H_26_O_10_	390.15260	[M‐H]‐1	390.15316	1.33	Loganin	389.14545, 227.09224, 127.03902, 101.02322	↓
10	8.36	C_23_H_38_O_4_	378.27701	[M+Na]+1	378.17780	1.08	2‐Arachidonoyl glycerol	378.28214, 195.12315, 154.96906	↓
11	9.28	C_18_H_32_O_2_	280.24023	[M‐H]‐1	280.24052	1.04	Linoleic acid	279.23325, 142.99539	↑
12	9.28	C_36_H_58_O_11_	666.39791	[M‐H]‐1	666.39416	2.59	1‐O‐[(2α,3β,19α)‐2,3,19,23‐Tetrahydroxy‐28‐oxoolean‐12‐en‐28‐yl]‐β‐D‐glucopyranose	666.39447, 504.34171, 409.31259	↓
13	9.88	C_6_H_13_O_9_P	260.02972	[M+H]+1	260.03004	1.22	Glucose 1‐phosphate	261.03735, 243.04982, 216.12328, 145.03925, 98.98475, 69.03433	↓
14	10.26	C_17_H_26_O_4_	294.18311	[M‐H]‐1	294.18361	1.69	6‐Gingerol	293.17633, 236.10522, 221.15421, 192.11513, 163.03923, 119.04906, 71.01251	↑
15	10.56	C_30_H_48_O_5_	488.35017	[M‐H]‐1	488.35090	1.48	Asiatic acid	511.34021, 489.34689, 453.33609, 425.34125, 407.33105, 249.18526, 221.15376, 205.15895, 191.14337, 145.10135, 95.08614	↑
16	10.87	C_9_H_8_O_3_	164.04734	[M+H]+1	164.04762	0.16	2‐Hydroxycinnamic acid	165.05489, 147.04422, 119.04955, 91.05489	↓
17	11.21	C_21_H_20_O_12_	464.09548	[M‐H]‐1	464.09638	1.32	Quercetin‐3β‐D‐glucoside	463.08911, 301.03580, 271.02521, 255.03021, 161.04457, 101.02312, 85.02803, 71.01240	↑
18	11.79	C_18_H_32_O_5_	328.22497	[M‐H]‐1	328.22545	1.44	Corchorifatty acid F	327.21817, 309.20703, 209.11781, 201.11214, 171.10196, 129.09102, 102.09589	↑
19	11.91	C_15_H_12_O_4_	256.07356	[M+H]+1	256.07396	2.67	4′,7‐Dihydroxyflavanone	257.08124, 183.10184, 165.09125, 137.05989105.07040	↓
20	11.92	C_16_H_22_O_10_	374.12135	[M+Na]+1	374.12171	1.09	Geniposidic acid	374.11414, 343.06744, 193.05002, 178.02638, 150.03125, 108.02045, 89.02309, 71.01249, 59.01248	↓
21	12.95	C_16_H_24_O_10_	376.13695	[M‐H]‐1	376.13727	0.85	Mussaenosidic acid	375.13, 337.05676, 213.07635, 169.08618, 151.07549, 125.05967, 89.02311, 71.01250, 59.01249	↑
22	13.38	C_15_H_10_O_7_	302.04265	[M‐H]‐1	302.04299	1.10	Quercetin	303.05026, 257.06027, 229.04959, 201.05486, 165.01843, 153.01843, 137.02359	↑
23	13.46	C_17_H_26_O_11_	451.14520	[M‐H]‐1	451.14581	1.37	Morroniside	451.14319, 405.14352, 243.08728, 179.05544, 155.03404, 141.05470, 101.02318, 89.02311, 59.01249	↓
24	15.23	C_15_H_12_O_7_	303.05051	[M‐H]‐1	303.0508	1.06	Dihydroquercetin	303.0402, 181.0153, 137.0241, 93.0328	↑
25	15.92	C_30_H_48_O_3_	456.36035	[M+H‐H2O]+1	456.36082	1.04	Ursolic acid	457.35126, 411.36261, 203.17989, 135.11693, 104.99279	↑
26	16.79	C_19_H_30_O_11_	434.17881	[M‐H]‐1	434.14326	−0.92	7‐O‐Ethylmorroniside	433.13599	↑
27	17.22	C_30_H_48_O_3_	456.36035	[M‐H]‐1	456.32014	2.59	Oleanolic acid	455.3535, 437.1653, 319.69037	↓
28	17.25	C_30_H_48_O_6_	504.34509	[M+H]+1	504.34541	0.63	Arjungenin	504.13016, 419.08194	↑
29	17.27	C_15_H_10_O_6_	286.04774	[M+H]+1	286.04808	1.20	Kaempferol	287.05536, 227.02133, 209.01096, 184.00325, 153.99254, 121.02885	↑
30	17.81	C_16_H_22_O_9_	358.12638	[M+H]+1	358.12645	0.23	Sweroside	359.13364, 197.08113, 179.07053, 126.03928, 111.08093, 97.02901, 85.02910	↑
31	17.90	C_7_H_6_O_4_	154.02661	[M‐H]‐1	154.02579	−5.34	Gentisic acid	153.01854, 138.03120, 123.00768, 110.98321, 95.01263, 66.99318	↑
32	18.19	C_7_H_12_O_6_	192.06339	[M‐H]‐1	192.06282	−1.54	Quinic acid	191.05554, 127.03900, 93.03330, 85.02819	↓
33	18.59	C_18_H_34_O_5_	330.24062	[M‐H]‐1	330.24107	1.36	(15Z)‐9,12,13‐Trihydroxy‐15‐octadecenoic acid	329.2338, 311.08154, 211.13367, 171, 10,191, 139.11182, 127.11155, 99.08028	↑
34	19.36	C_18_H_36_O_2_	284.27153	[M‐H]‐1	284.27199	1.61	Stearic acid	283.26471, 239.03482	↑
35	21.16	C_18_H_34_O_2_	282.25588	[M‐H]‐1	282.25627	1.38	Oleic acid	281.24899, 239.3490	↓
36	21.77	C_6_H_5_NO_2_	123.032034	[M+H]+1	123.03246	3.49	Nicotinic acid	124.03973, 96.04496, 80.05019	↓
37	22.19	C_5_H_13_NO	103.09971	[M+H]+1	103.10021	4.70	Choline	104.10748, 87.04471, 60.08165	↓
38	22.44	C_17_H_24_O_10_	433.13463	[M‐H]‐1	434.14274	0.54	Verbenalin	433.13540, 387.12918, 226.08000, 225.07661, 123.04411, 101.02322, 68.99689	↑
39	22.48	C_8_H_8_O_5_	184.03717	[M‐H]‐1	184.03661	−3.09	Methyl gallate	184.03278, 165.05618, 139.03908, 111.000760, 97.02824, 71.4892	↑
40	22.92	C_14_H_6_O_8_	302.00627	[M‐H]‐1	302.00676	1.63	Ellagic acid	300.99948, 229.01398, 185.02383	↑
41	24.12	C_9_H_6_O_4_	178.02661	[M+H]+1	178.02704	1.24	7,8‐Dihydroxycoumarin	179.03432, 161.05995, 147.04424, 133.06505, 111.04455, 105.07043, 81.07063	↑
42	26.91	C_22_H_18_O_12_	474.07983	[M‐H]‐1	474.08079	0.14	Cichoric acid	473.09418, 341.08804, 311.06247, 197.04506, 179.03430, 135.04413, 89.02313, 59.01252	↑

### 3.6 pCO Attenuates Joint Pain and Swelling in OA Rats

**FIGURE 8:**
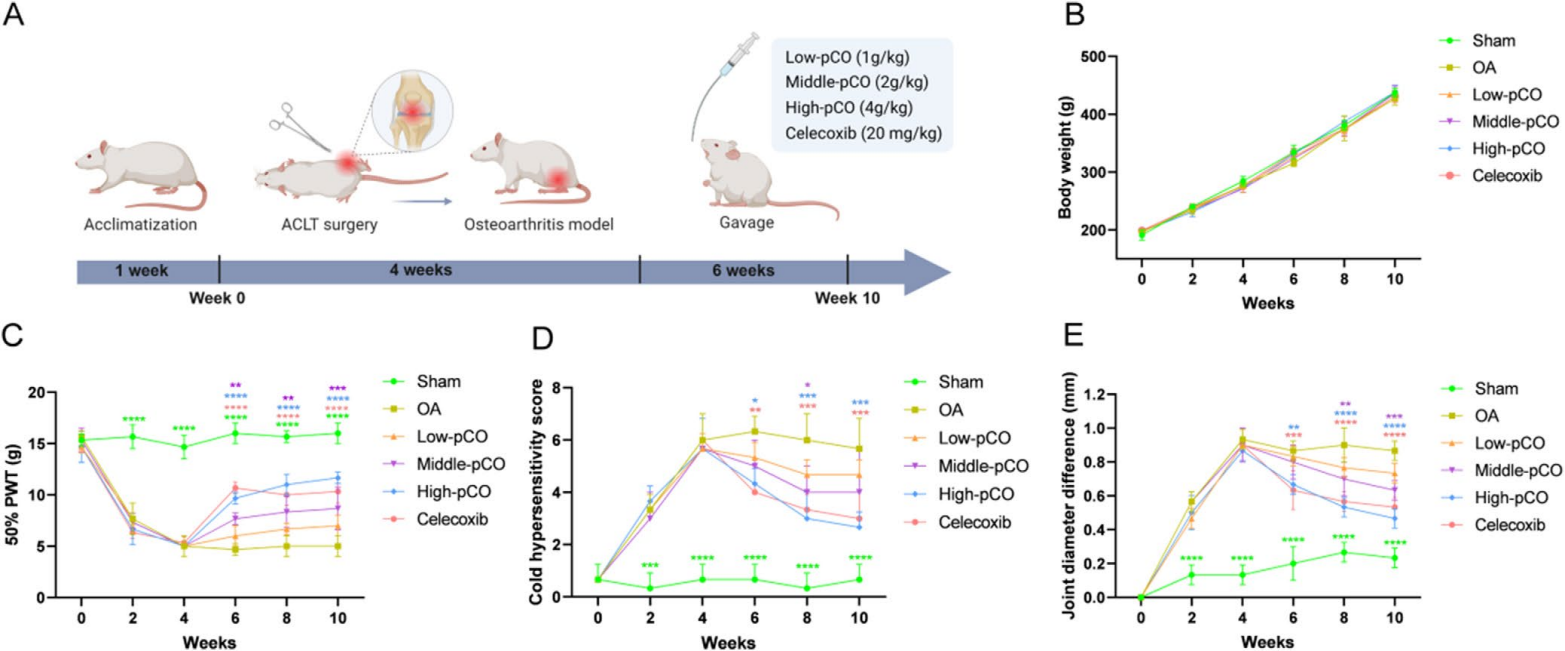
Effect of pCO on alleviating joint damage and pain in OA rats after ACLT surgery. (A) Schematic diagram of the time course of ACLT‐induced knee joint damage in OA rats. (B) Body weight change curve of rats. (C) Mechanical pain detection curve. (D) Cold sensitivity test score curve. (E) Knee joint width measurement curve. *p < 0.05 versus OA group, **p < 0.01 versus OA group, ***p < 0.001 versus OA group, ****p < 0.0001 versus OA group.

Figure 8A showed the experimental design and timeline of the ACLT‐induced OA rat model. During the experimental period, body weight gradually increased in all groups, with no statistically significant differences observed among groups (Figure 8B). Beginning after model establishment, paw withdrawal threshold (PWT), cold sensitivity score and joint swelling were assessed in each group through behavioural tests. Prior to drug intervention, significant differences were observed between the sham group and the OA group in PWT, cold sensitivity score and joint swelling, whereas no statistically significant differences were found between the OA group and the drug intervention groups (Figure 8C–E). After 6 weeks of drug intervention, the OA group exhibited a significant decrease in PWT, along with significant increases in cold sensitivity score and joint swelling. In contrast, the middle‐pCO, high‐pCO and celecoxib groups showed significantly improved PWT and markedly reduced joint swelling. Cold sensitivity scores were significantly decreased in the high‐pCO and celecoxib groups. These findings demonstrate that pCO and celecoxib alleviate cold sensitivity and joint swelling in ACLT‐induced OA rats, with the high‐pCO group exhibiting the most marked improvement in PWT, joint pain and swelling.

Furthermore, 6 weeks of oral high‐dose pCO administration did not significantly alter the histopathology of major organs (liver, kidney and lung) or liver/kidney function (AST, ALT, Cr, BUN) compared to the sham group (Figure S1).

### 3.7 pCO Ameliorates Articular Cartilage Damage

**FIGURE 9:**
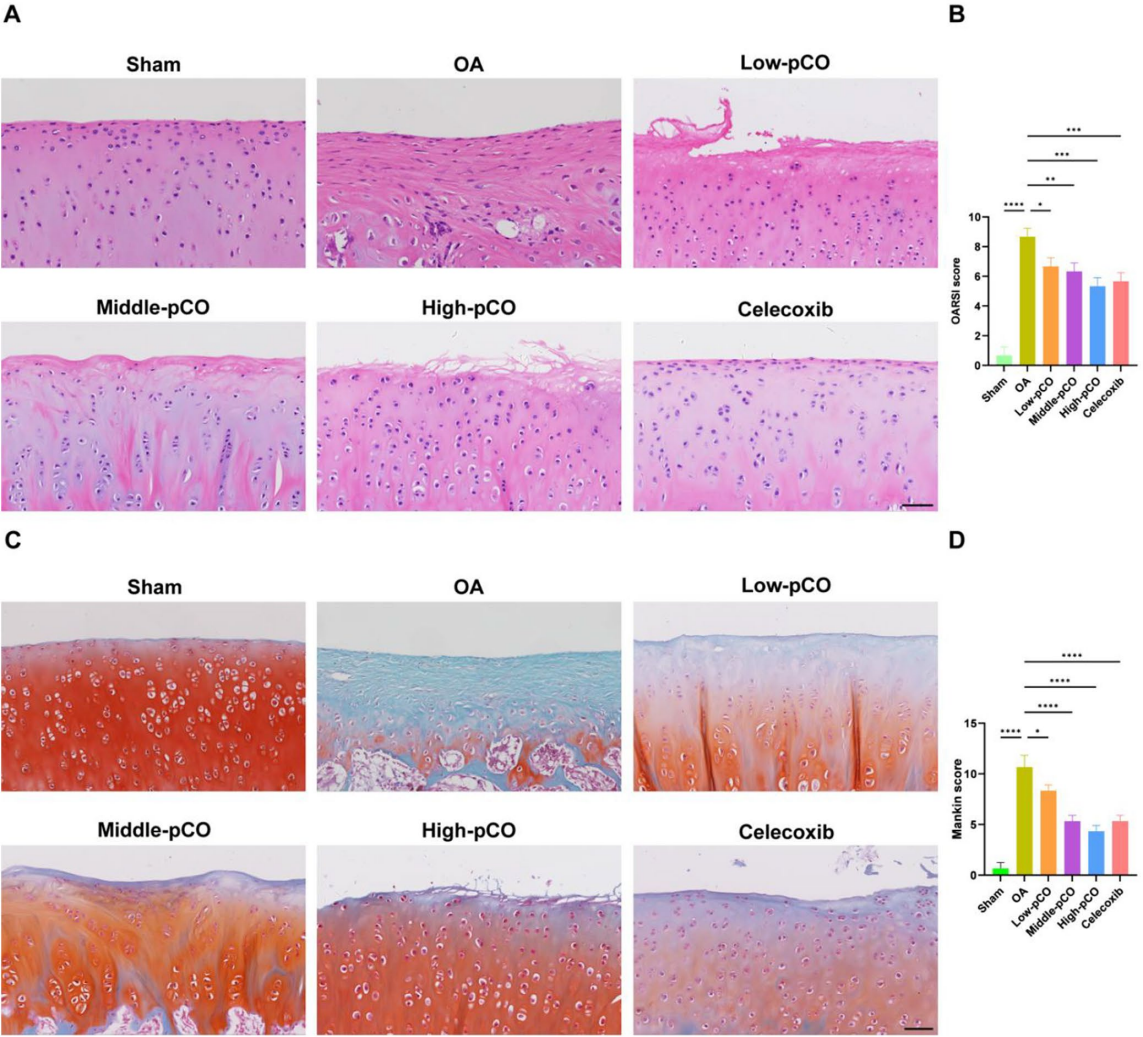
Histopathological staining analysis demonstrated pCO‐mediated amelioration of cartilage degeneration in OA rats. (A) H&E staining of cartilaginous matrix. Scale bar = 100 μm. (B) OARSI score of knee joints in OA rats. (C) S‐O staining of cartilaginous matrix. Scale bar = 100 μm. (D) Mankin score in OA rats. Data is expressed as mean ± SD (*n* = 6). *p < 0.05 versus OA group, ****p < 0.0001 versus OA group.

After six weeks of treatment by oral gavage, histopathological changes of the rats were assessed by H&E and S‐O staining (Figure 9A,C). H&E staining revealed the smooth cartilage surface, intact structure and normal chondrocyte morphology in the sham group. In contrast, the OA group exhibited destruction of the superficial cartilage layer, characterized by a reduced number and irregular distribution of chondrocytes. The pCO and celecoxib groups showed relative amelioration of cartilage matrix, with more homogeneous matrix staining and increased, neatly distributed chondrocytes. S‐O staining further demonstrated the results, showing severe extracellular matrix damage and irregular cartilage surface morphology in the OA group, while the pCO and celecoxib groups exhibited differential improvement in cartilage matrix. The effects were quantitatively evaluated using OARSI and Mankin scoring systems (Figure 9B,D). The OA group exhibited significantly higher scores compared to the sham group. Conversely, pCO and celecoxib groups significantly reduced these scores compared to the OA group, with the high pCO group exhibiting the most pronounced improvement.

### 3.8 pCO Ameliorates Synovitis and Synovial Fibrosis

**FIGURE 10:**
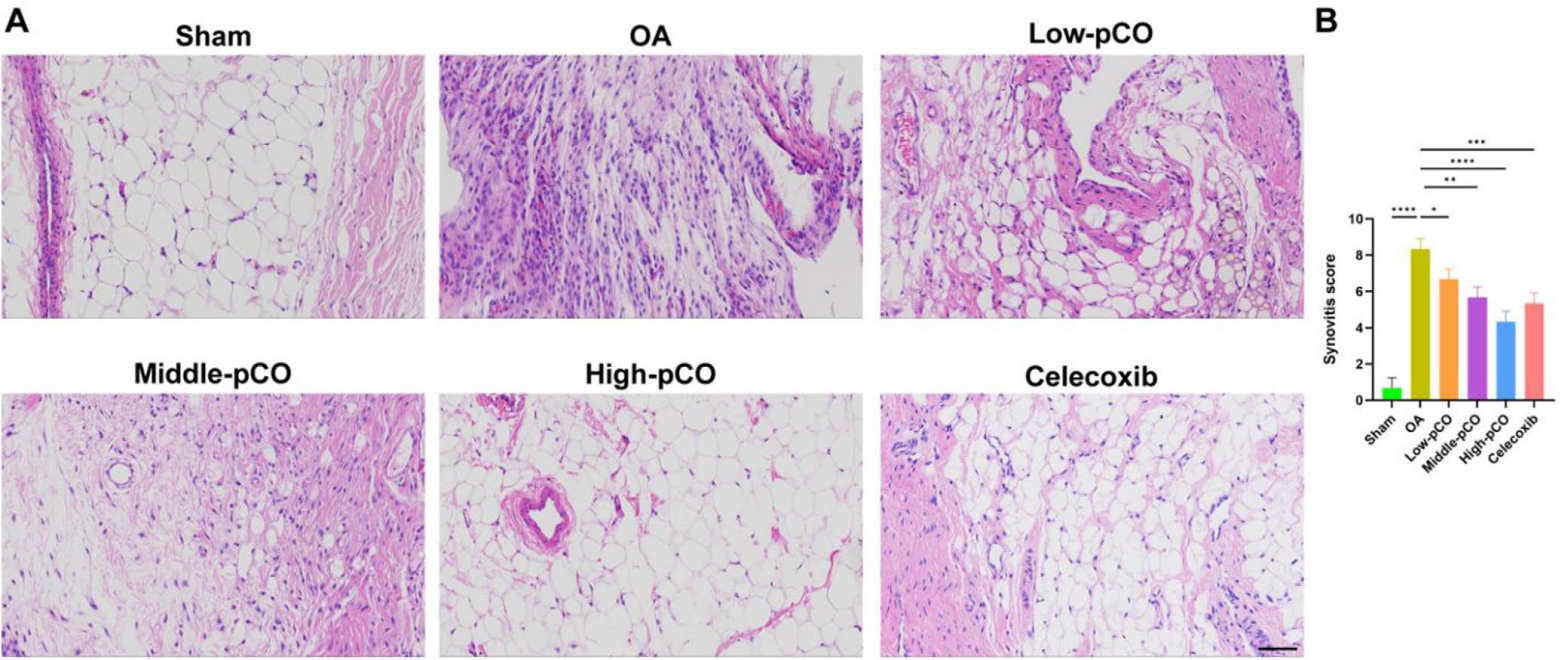
The administration of pCO attenuated synovial inflammatory responses in OA rats. (A) H&E staining of synovium in rats. Scale bar = 100 μm. (B) The synovitis score in rats. Data is expressed as mean ± SD (*n* = 6). *p < 0.05 versus OA group, **p < 0.01 versus OA group, ***p < 0.001 versus OA group, ****p < 0.0001 versus OA group.

The effect of pCO treatment on synovial inflammation in OA rats was assessed by H&E staining of the knee joint synovium (Figure 10A). In the OA group, the synovial membrane exhibited structural disorganization, characterized by inflammatory cell infiltration, abnormal hyperplasia, vascular congestion and synovial fibrosis. Compared to the OA group, the pCO and celecoxib groups demonstrated significant amelioration of synovitis, with the high‐pCO group exhibiting the most marked reduction in synovitis scores (Figure 10B).

### 3.9 pCO Attenuates Articular Cartilage Degeneration and Apoptosis

**FIGURE 11:**
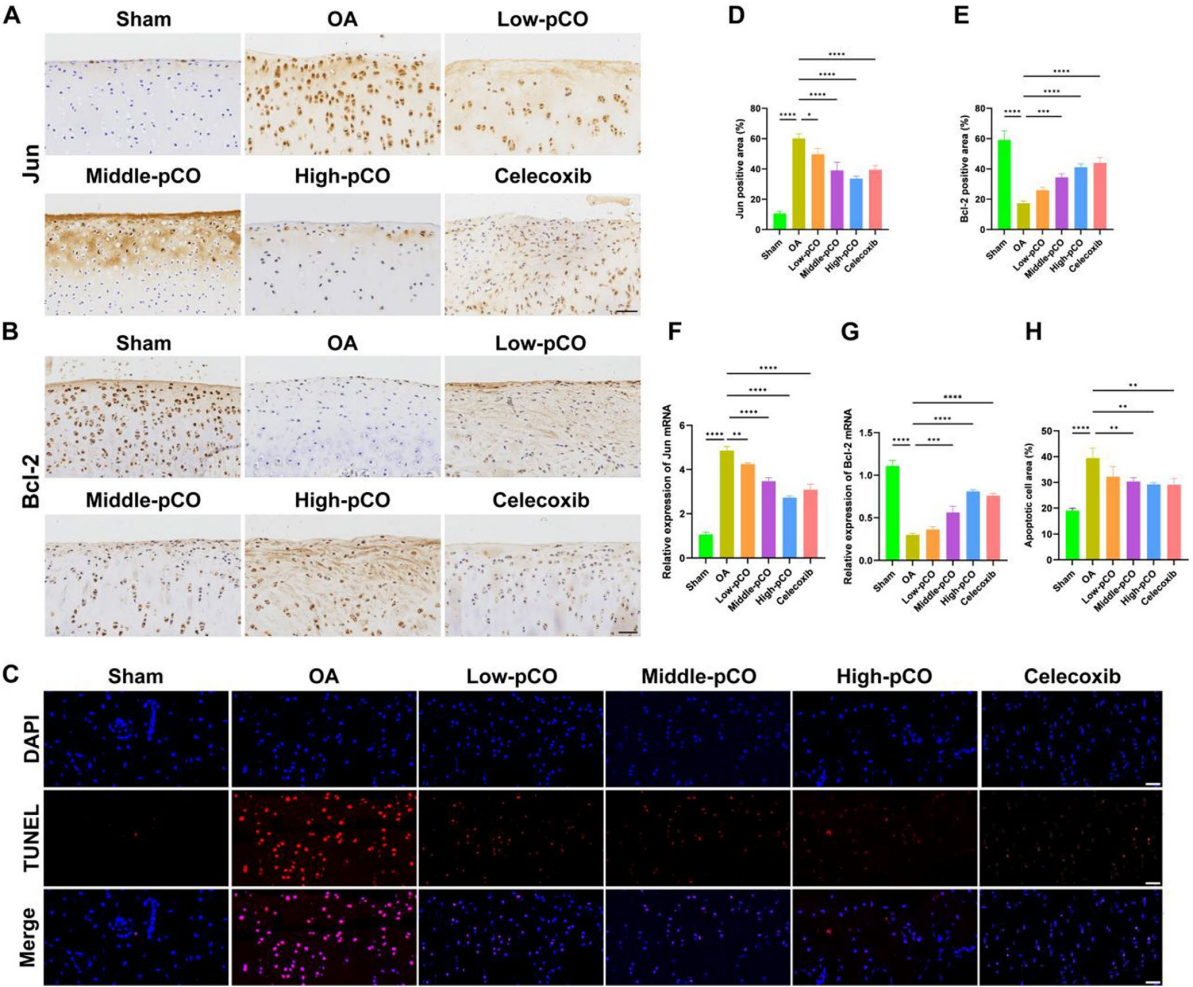
The regulation of pCO on cartilage degeneration and apoptosis. (A, B) Immunohistochemical staining of Jun and Bcl‐2 in articular cartilage. Scale bar = 100 μm. (C) The TUNEL assay showed blue fluorescence of DAPI, red fluorescence of TUNEL and finally the merged plot. Scale bar = 20 μm. (D, E) Quantitative analysis of Jun and Bcl‐2 immunohistochemical staining. (F, G) Relative mRNA expression levels of Jun and Bcl‐2 in cartilage tissue determined by RT‐qPCR. (H) Quantitative analysis of apoptotic chondrocytes by TUNEL assay. Data is expressed as mean ± SD (*n* = 6). *p < 0.05 versus OA group, ***p < 0.001 versus OA group, ****p < 0.0001 versus OA group.

Immunohistochemistry and RT‐qPCR were performed to assess the expression of Jun and Bcl‐2 in the articular cartilage. In the OA group, the immunohistochemistry expression and mRNA levels of Jun were significantly increased. Conversely, therapeutic intervention with pCO and celecoxib decreased Jun expression in articular cartilage (Figure 11A,D,F). In the OA group, the immunohistochemistry and mRNA expressions of Bcl‐2 were significantly reduced. Compared to the OA group, high‐pCO and celecoxib groups significantly increased Bcl‐2 protein expression and mRNA levels. Among the pCO groups, the high‐pCO group exhibited the most significant anti‐apoptotic effect (Figure 11B,E,G).

To further evaluate the anti‐apoptotic effect of pCO, TUNEL staining was performed. Quantitative analysis revealed a significant increase in both the area and number of TUNEL‐positive cells in the OA group, indicating enhanced chondrocyte apoptosis during OA progression. Compared to the OA group, both the pCO and celecoxib groups exhibited significantly reduced chondrocyte apoptosis. It is noteworthy that the high‐pCO group exhibited the most pronounced suppressive effect on chondrocyte apoptosis (Figure 11C,H).

### 3.10 pCO Modulates Inflammatory Factor Levels in Serum

**FIGURE 12:**
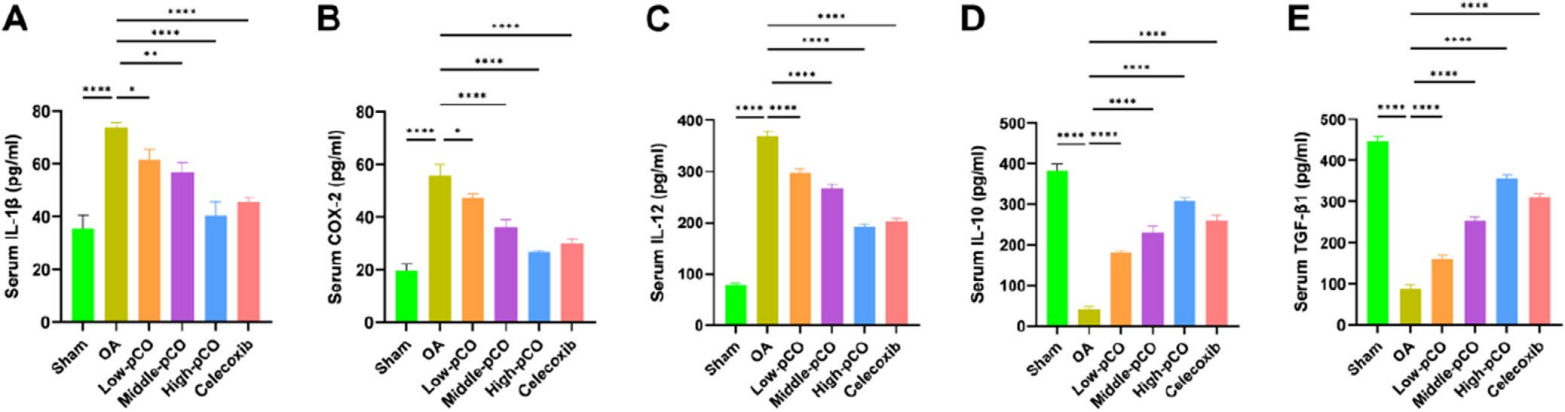
Effects of pCO on serum inflammatory cytokine levels in OA rats. Serum concentrations of (A) IL‐1β, (B) COX‐2, (C) IL‐12, (D) IL‐10 and (E) TGF‐β1. Data is expressed as mean ± SD (*n* = 6). *p < 0.05 versus OA group, **p < 0.01 versus OA group, ****p < 0.0001 versus OA group.

In the OA group, serum levels of the pro‐inflammatory factors IL‐1β, COX‐2 and IL‐12 were significantly elevated (Figure 12A–C), while the anti‐inflammatory factors IL‐10 and TGF‐β1 were markedly reduced compared to the sham group (Figure 12D,E). Treatment with pCO dose‐dependently reversed these changes, significantly decreasing IL‐1β, COX‐2 and IL‐12 levels and increasing IL‐10 and TGF‐β1 levels relative to the OA group. The high‐pCO group showed the most pronounced modulation of serum pro‐inflammatory and anti‐inflammatory factor levels.

### 3.11 pCO Modulates Inflammatory Factor Levels in Synovial Fluid

**FIGURE 13:**
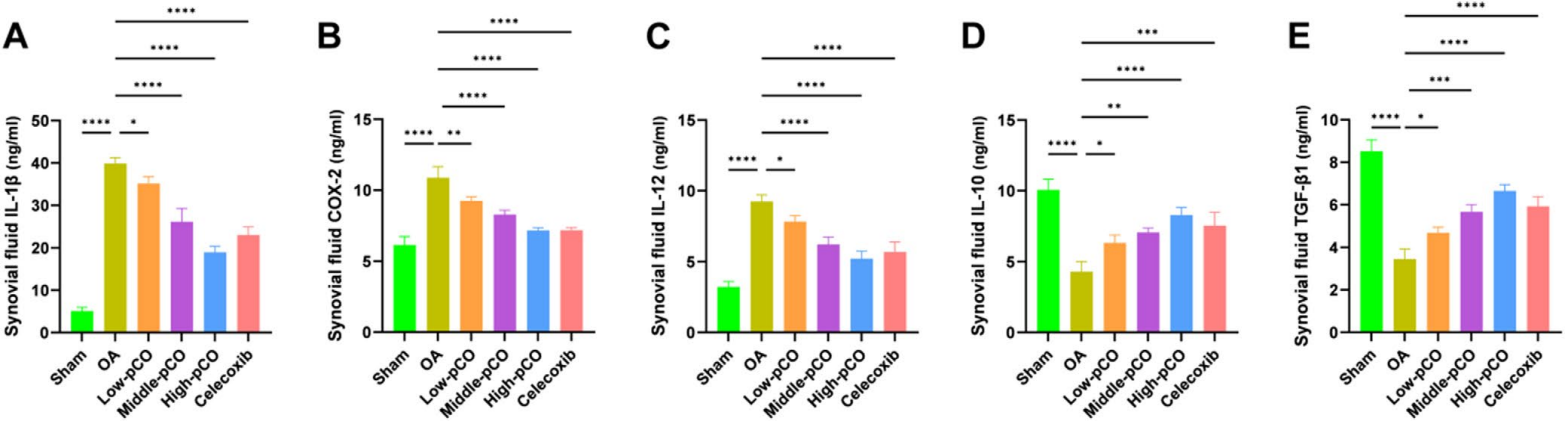
Effects of pCO on synovial fluid inflammatory cytokine levels in OA rats. Synovial fluid concentrations of (A) IL‐1β, (B) COX‐2, (C) IL‐12, (D) IL‐10 and (E) TGF‐β1. Data is expressed as mean ± SD (*n* = 6). *p < 0.05 versus OA group, **p < 0.01 versus OA group, ***p < 0.001 versus OA group, ****p < 0.0001 versus OA group.

As shown in Figure 13, the levels of pro‐inflammatory factors IL‐1β, COX‐2 and IL‐12 in synovial fluid were significantly elevated in OA rats compared to the sham group. Treatment with pCO or celecoxib markedly reduced these cytokine levels relative to the OA group. Conversely, the anti‐inflammatory factors IL‐10 and TGF‐β1 were significantly lower in the synovial fluid of OA rats compared to the sham groups. The high‐ pCO group exhibited the most pronounced modulatory effect on both pro‐inflammatory and anti‐inflammatory cytokine levels.

### 3.12 pCO Upregulates TGF‐β1 Expression to Ameliorate the OA Environment

**FIGURE 14:**
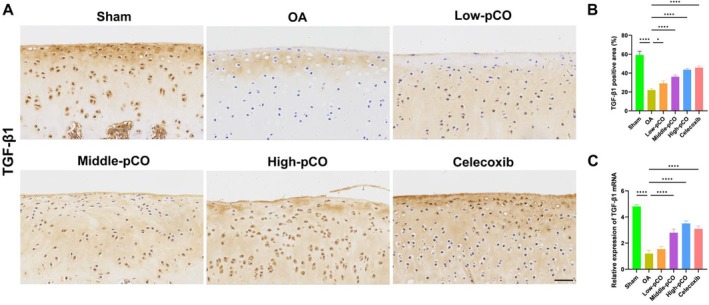
pCO improved the intra‐articular inflammatory environment. (A) Positive expression of TGF‐β1 was examined in articular cartilage. Scale bar = 100 μm. (B) Immunohistochemical quantitative analysis of TGF‐β1. (C) mRNA levels of TGF‐β1 in synovial tissue. Data is expressed as mean ± SD (*n* = 6). *p < 0.05 versus OA group, **p < 0.01 versus OA group, ****p < 0.0001 versus OA group.

Immunohistochemistry was performed to evaluate TGF‐β1 expression and its anti‐inflammatory effects in the osteoarthritic microenvironment. TGF‐β1 expression was significantly reduced in OA rat models, while pCO administration markedly increased TGF‐β1 levels in a dose‐dependent manner. In addition, the mRNA level of TGF‐β1 in the synovium was significantly decreased in the OA group relative to the sham group. The pCO and celecoxib groups significantly increased TGF‐β1 expression compared to the OA group, with the high‐pCO group exhibiting the most significant efficacy (Figure 14).

### 3.13 pCO Modulates M1/M2 Macrophage Polarization in the Synovium of OA Rats

**FIGURE 15:**
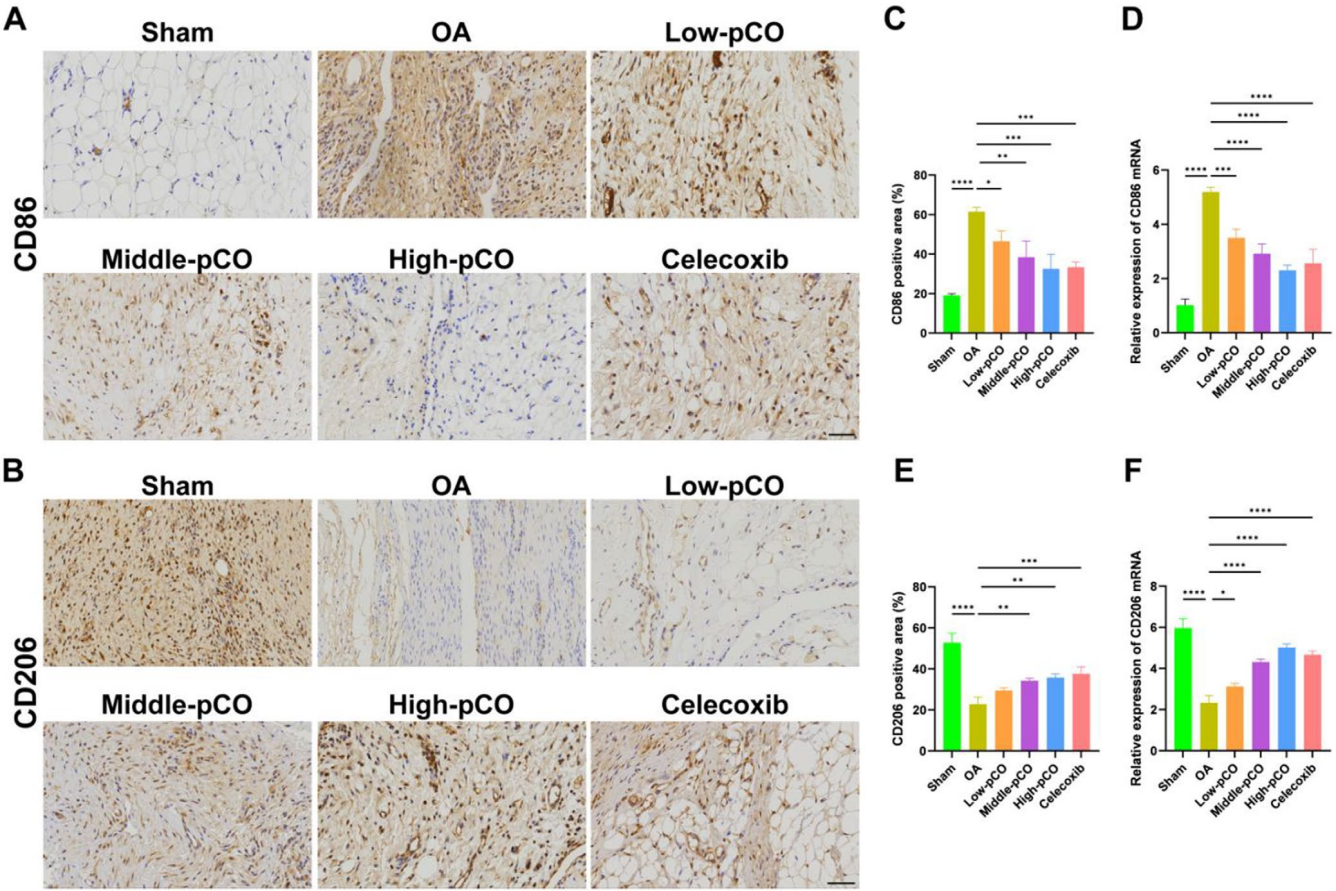
Immunohistochemical staining of pCO‐regulated synovial macrophages in OA. (A) Expression of CD86 (M1 phenotype) positive expression in synovial tissue. Scale bar = 100 μm. (B) Expression of CD206 (M1 phenotype) positive expression in synovial tissue. (C) Quantitative analysis of CD86‐positive expression in synovial tissue. Scale bar = 100 μm. (D) mRNA expression of CD86 in synovial tissue. (E) Quantitative analysis of CD206‐positive expression in synovial tissue. (F) mRNA expression of CD206 in synovial tissue. Data is expressed as mean ± SD (*n* = 6). *p < 0.05 versus OA group, **p < 0.01 versus OA group, ***p < 0.001 versus OA group, ****p < 0.0001 versus OA group.

Immunohistochemical analysis of synovial macrophage phenotypes was performed to evaluate the effect of pCO on macrophage expression in OA rats (Figure 15A,B). Compared to the sham group, the OA group exhibited increased positive expression and mRNA levels of CD86 (M1 phenotype), along with decreased positive expression and mRNA levels of CD206 (M2 phenotype) (Figure 15C,D). Following pCO treatment, CD86 expression was reduced, while the positive expression and mRNA levels of CD206 were significantly enhanced (Figure 15E,F).

**FIGURE 16:**
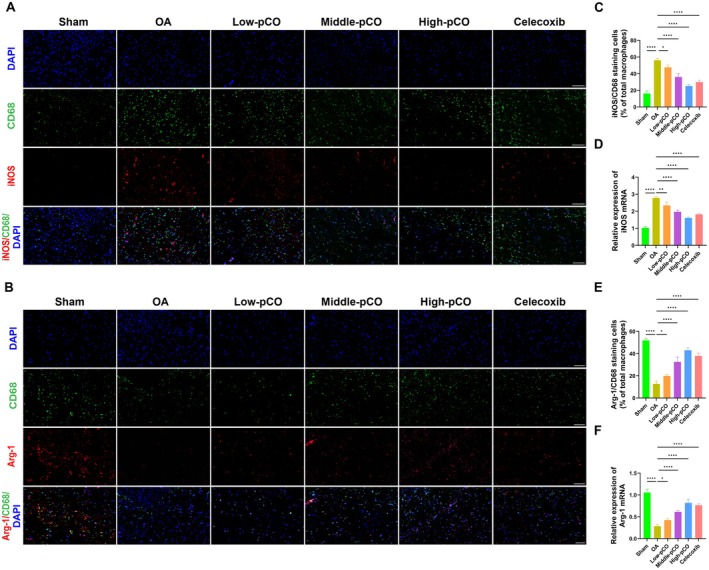
pCO modulates M1/M2 macrophage polarization in the synovium of OA rats. (A) Double labeling of iNOS (M1) and CD68 (pan‐macrophage). Scale bar = 100 μm. (B) Double labeling of Arg‐1 (M2) and CD68 (pan‐macrophage). Scale bar = 100 μm. (C) Quantitative analysis of iNOS/CD68 positive expression in the synovium. (D) Relative mRNA expression of iNOS in synovial tissue. (E) Quantitative analysis of Arg‐1/CD68 positive expression in the synovium. (F) Relative mRNA expression of Arg‐1 in synovial tissue. Data is expressed as mean ± SD (*n* = 6). *p < 0.05 versus OA group, ***p < 0.001 versus OA group, ****p < 0.0001 versus OA group.

Immunofluorescence analysis was performed to explore the molecular mechanism underlying the immunomodulatory function of pCO on synovial macrophages. CD68 was employed as a pan‐macrophage marker, while iNOS (M1 phenotype) and Arg‐1 (M2 phenotype) were used to identify pro‐inflammatory and anti‐inflammatory macrophages, respectively (Figure 16A,B). The protein expression of iNOS was significantly increased in the OA group, while it was markedly reduced in the pCO and celecoxib groups (Figure 16C). The mRNA expression level of iNOS was significantly elevated in the OA group, whereas it was significantly decreased in the pCO and celecoxib groups (Figure 16D). In addition, the protein expression of Arg‐1 was significantly decreased in the OA group compared to the sham group, while it was notably increased in the pCO and celecoxib groups (Figure 16E). The mRNA level of Arg‐1 was significantly reduced in the OA group, while it was significantly increased in the pCO and celecoxib groups (Figure 16F). Among the pCO groups, the high‐pCO group showed the most significant improvement.

## 4 Discussion

The current scarcity of therapeutic strategies for OA has catalysed growing interest in exploring specific herbs as an adjunctive treatment modality [47]. A previous study has demonstrated that CO‐related extracts reduce inflammation and joint destruction during OA progression [48]. However, the role of its processed product, pCO, in the treatment of OA and its underlying mechanisms remains unclear. The present study revealed that pCO alleviates joint pain, swelling and degeneration during OA progression, particularly through the biological mechanism of regulating M1/M2 macrophage polarization. Our results indicated that pCO administration significantly attenuated cartilage degeneration and synovitis in ACLT‐induced OA rats. Decreased Jun expression and increased Bcl‐2 expression in cartilage tissue suggested that pCO possesses the potential to alleviate cartilage matrix degradation and exert anti‐apoptotic effects. pCO administration suppressed the levels of IL‐1β, COX‐2 and IL‐12 in serum and synovial fluid, while significantly increasing IL‐10 and TGF‐β1 levels, indicating that pCO reduces pro‐inflammatory factors and enhances anti‐inflammatory effects. Furthermore, the increased expression of TGF‐β1 further suggested that pCO enhances anti‐inflammatory effects in articular cartilage and ameliorates the inflammatory and degradative environment within cartilage. Following pCO treatment, the expression of pro‐inflammatory markers CD86 and iNOS (M1 phenotype) decreased in the OA synovium, whereas the expression of anti‐inflammatory markers CD206 and Arg‐1 (M2 phenotype) increased. This shift in the M1/M2 ratio indicated that pCO possesses the potential to regulate M1/M2 macrophage polarization in the synovium of OA rats. In the modulation of M1/M2 macrophage polarization by pCO intervention, high‐dose pCO appeared to exhibit the most optimal regulatory efficacy in OA rats. Therefore, pCO demonstrated a significant therapeutic effect against OA, and maintaining an appropriate dosage range is of great importance.

HPLC‐Q‐Orbitrap‐MS analysis was employed to compare the compositional changes between raw CO and pCO, clarifying the differences in their chemical constituents and the potential therapeutic effects arising from these differences. In this study, 42 differential components were identified in CO and pCO by comparing MS/MS characteristic spectra with reference standards and relevant literature. Multiple algorithms, including PCA‐X and OPLS‐DA, were applied to rigorously validate the differences between CO and pCO. Among these components, changes in flavonoids, terpenoids and phenolic acids, such as kaempferol, asiatic acid, loganin, gallic acid, gingerol and ellagic acid, have been demonstrated in previous studies to possess significant anti‐inflammatory and anti‐degenerative activities. Kaempferol has been shown to exert anti‐inflammatory and anti‐apoptotic effects, reduce cartilage catabolism and promote chondrogenesis of ADMSCs [49, 50]. Yang et al. found that asiatic acid effectively inhibited the pro‐inflammatory cytokine levels of iNOS and COX‐2, and promoted the expression of glycosaminoglycans (GAGs) and type II collagen (Col II) to alleviate cartilage degeneration and destruction [51]. Wan et al. reported that loganin significantly reduced IL‐1β‐mediated mRNA expression of PGE2, NO, iNOS and COX‐2 in chondrocytes, and suppressed synovial fluid inflammation [52]. Hernández‐Valencia et al. demonstrated that gallic acid reduced pro‐inflammatory cytokine levels of IL‐6, TNF‐α and IL‐1β in human OA synovial cells [53]. Gallic acid has also been shown to reduce IL‐1β, IL‐6 and TNF‐α levels in synovial cells, alleviating inflammatory infiltration and fibrous tissue hyperplasia in the synovium by altering gut microbiota abundance [54]. Ma et al. found that gingerol promoted extracellular matrix (ECM) regeneration in chondrocytes and attenuated oxidative stress, inflammation and apoptosis [55]. Zahran et al. reported that ursolic acid significantly inhibited serum levels of TNF‐α, COX‐2, IL‐6 and IL‐1β in rats, thereby modulating immune and inflammatory responses and slowing OA chondrocyte degeneration [56]. Qu et al. demonstrated that cichoric acid significantly suppressed TNF‐α‐induced overproduction of PGE2, iNOS, COX‐2 and IL‐12 in human C28/I2 chondrocytes, and downregulated ADAMTS‐5 and matrix metalloproteinases (MMPs) to alleviate ECM degradation [57]. Our study found that serum levels of IL‐1β, COX‐2 and IL‐12 were increased in the OA group rats, with significantly greater joint pain and cartilage degeneration compared to the sham group, findings that are consistent with previous studies by Park et al. [58]. Chen et al. discovered that ellagic acid inhibited COX‐2 and PGE2 production in mouse serum and suppressed M1 macrophage polarization to ameliorate OA progression [59]. In this study, following 6 weeks of pCO treatment, serum assays revealed not only a reduction in pro‐inflammatory cytokine secretion but also an increase in the expression levels of anti‐inflammatory factors IL‐10 and TGF‐β1. Combined with the analysis results of synovial fluid, pCO was shown to promote the synthesis of anti‐inflammatory factors and alleviate joint pain, swelling and cartilage damage.

PPI network topology analysis in bioinformatics elucidated the pharmacological targets of pCO in OA treatment. KEGG enrichment analysis identified the apoptosis pathway as a significant signalling pathway. Molecular docking simulations were performed to validate the binding interaction between pCO and OA. Bcl‐2, a key anti‐apoptotic protein, is associated with the inhibition of Bax, protecting chondrocytes from apoptosis [60]. High expression of Bcl‐2 forms heterodimers with Bax, inhibiting Bax translocation and dimerization, blocking Cyt‐c release and effectively suppressing apoptotic activity [61]. In this study, the OA group exhibited decreased mRNA and immunohistochemical expression of Bcl‐2, whereas pCO treatment significantly increased Bcl‐2 expression in OA rat cartilage and reduced chondrocyte apoptosis, as confirmed by TUNEL assay. Jun, a transcription factor, recognizes and binds with heterologous FOS family proteins to form the AP‐1 transcription complex [62]. Jun can lead to MMP overexpression, thereby accelerating OA cartilage matrix degradation and damage [63]. Our findings demonstrate that pCO reduced Jun mRNA expression and immunohistochemical staining, thereby alleviating cartilage degeneration.

Different innate and adaptive immune cells participate in the inflammation and disease progression of OA, affecting all joint components, including the synovium, synovial fluid and superficial and deep cartilage layers [64]. Synovial macrophages are key immune cells involved in synovial inflammation and exhibit both pro‐inflammatory and anti‐inflammatory phenotypes [65]. Dysfunction of synovial macrophages can disrupt the balance between pro‐inflammatory and anti‐inflammatory factors, thereby inducing synovial fibrosis and inflammatory hyperplasia [66]. M1 macrophage polarization releases pro‐inflammatory mediators, including IL‐1β and COX‐2, inducing the synthesis of cartilage matrix‐degrading enzymes and intra‐articular inflammation in OA [67]. In this study, ACLT‐induced OA resulted in increased M1 macrophage abundance and pro‐inflammatory cytokine expression in synovial fluid compared to the sham group, consistent with the OA joint characteristics reported by Boyer et al. [68]. IL‐10 and TGF‐β1, anti‐inflammatory factors secreted by M2 macrophages, play essential roles in inhibiting synovial fibrosis and cartilage‐destructive proteins [69]. Li et al. demonstrated that upregulation of key anti‐inflammatory mediators in synovial fluid enhanced macrophage polarization toward the anti‐inflammatory and pro‐repair M2 phenotype, attenuating synovial fibrosis and inflammatory damage in OA rats [70]. Our study showed that pCO reduced pro‐inflammatory factor expression in synovial fluid and promoted increased expression of IL‐10 and TGF‐β1 to alleviate synovitis. Synovial fluid serves as a critical medium for metabolite exchange during joint inflammation and cartilage regeneration, and shedding of inflammatory chondrocytes into synovial fluid can cause secondary damage to the synovium, exacerbating cartilage destruction [71]. This study supported that pCO promotes high expression of TGF‐β1 in the synovium and cartilage, significantly improving the anti‐inflammatory environment within the joints of OA rats.

Imbalance in the M1/M2 macrophage ratio is associated with inflammatory progression in OA [72]. Restoring the M1/M2 macrophage balance is considered a novel therapeutic approach, particularly for inflammatory and metabolic phenotypes of OA [73]. In this study, immunohistochemical results showed that the expression level of the M1 marker (CD86) was significantly higher in the OA group compared to the sham group, whereas the expression level of the M2 marker (CD206) was significantly reduced. These findings were consistent with RT‐qPCR results. A previous report demonstrated that CO and its related components suppressed M1‐type pro‐inflammatory markers in RAW 264.7 macrophages [74], but this phenomenon has not been previously reported for pCO in OA. In this study, pCO intervention not only reduced the immunohistochemical and gene expression levels of M1 macrophages but also, notably, significantly increased the expression levels of M2 markers. CD68 is widely used as a pan‐macrophage marker in the synovium of OA rats [75]. M1 macrophages release inflammatory mediators (iNOS) that exacerbate inflammatory responses and tissue damage [76]. As inflammatory stimuli diminish or anti‐inflammatory factors such as IL‐10 increase, macrophages are polarized toward the M2 anti‐inflammatory phenotype [77]. M2 macrophages shift their metabolic pathway toward oxidative phosphorylation and release Arg‐1 and TGF‐β1 to promote tissue repair and alleviate damage [78]. Our results demonstrated that pCO increased the expression of the M2 marker (Arg1), significantly reduced the number of iNOS/CD68‐positive cells, and significantly increased the number of Arg1/CD68‐positive cells. Xu et al. found that promoting the transition of M1 macrophages to the M2 phenotype reduced inflammatory factor release, thereby alleviating OA inflammation and pain [79]. Klyucherev and colleagues discovered that targeting reduced iNOS expression and increased Arg‐1 expression in macrophages holds significant potential for regulating inflammation and promoting joint tissue regeneration in OA [80]. These findings suggested that pCO ameliorates OA inflammation by promoting M2 macrophage polarization in the rat synovium.

In conclusion, this study demonstrates that pCO may inhibit inflammatory responses, exert chondroprotective effects and ameliorate joint pain and swelling in OA rats by modulating M1/M2 macrophage polarization. The differential component changes revealed by HPLC‐Q‐Orbitrap‐MS highlight the potential key constituents of pCO in the treatment of OA. However, our experimental investigation into the mechanisms by which pCO improves OA in rats has only uncovered the tip of the iceberg. We plan to conduct more in‐depth mechanistic studies based on serum metabolomics, for instance, further identifying the compositional changes between CO and pCO and incorporating molecular docking to screen whether key components exhibit superior immunomodulatory effects on synovial cells or chondrocytes. Future flow cytometry identification is also required to thoroughly distinguish between synovial resident macrophages and peripherally derived differentiated macrophages/monocytes, thereby comprehensively elucidating its pharmacological mechanism. Furthermore, additional validation experiments using target inhibitors or gene knockout rats are necessary to confirm how pCO protects articular cartilage tissue. Based on network pharmacology analysis results, TGF‐β1 exhibited favourable molecular docking outcomes, and combined with experimental findings, it appears to be an important mechanism through which pCO exerts therapeutic effects in OA. TGF‐β1 targets multiple factors regulating chondrocytes and improving joint inflammation, including Smad1, Smad2/3 and MAPK6. A recent study demonstrated that TGF‐β1‐stimulated miR‐135b promotes M2 macrophage polarization by targeting rat bone marrow mesenchymal stem cells (BMSCs), thereby ameliorating cartilage damage, providing a novel regulatory mechanism for OA treatment [81]. Similarly, a critical link exists between the paracrine effects of M2 macrophages and the activation of TGF‐β‐induced Smad2/3 phosphorylation. Coordinated regulation of this pathway may promote chondrocyte regeneration and ameliorate human chondrocyte injury [82]. Therefore, the profound mechanism of TGF‐β1 in M2 macrophage polarization mediated by pCO in OA treatment warrants further investigation.

## 5 Conclusion

The present study confirmed that pCO exhibits therapeutic effects against OA. pCO alleviated joint pain, swelling and cartilage degeneration, and attenuated synovial lesions to ameliorate cartilage damage in an OA rat model. Network pharmacology and HPLC‐Q‐Orbitrap‐MS were employed to identify potential biological mechanisms and key differential components. Accordingly, pCO exerted anti‐inflammatory and immunomodulatory effects in OA by regulating M1/M2 macrophage polarization. This study demonstrated the therapeutic potential of pCO in anti‐OA and provided theoretical support for its application. However, further experiments are still required to fully elucidate the underlying mechanisms.

## Author Contributions

**Yongsheng Fu:** conceptualization (lead); data curation (lead); methodology (lead); software (lead); writing – original draft (lead). **Minghua Zhao:** conceptualization (equal); data curation (equal); methodology (equal); writing – review and editing (equal). **Xudong Huang:** data curation (equal); methodology (equal); software (equal). Yungang Wu: conceptualization (equal); data curation (equal). **Yingchao Ren:** data curation (equal); validation (equal). **Weiguo Wang:** conceptualization (lead); funding acquisition (lead); methodology (lead); supervision (lead); writing – review and editing (lead).

## Funding

This work was supported by the Clinical Research Project of the Wu Jieping Medical Foundation (No. 320.6750.18548).

## Ethics Statement

This study was approved by the Animal Experiment Ethics Committee of Shandong University of Traditional Chinese Medicine Affiliated Hospital (Animal Experimentation Ethics No. AWE‐2023‐007).

## Conflicts of Interest

The authors declare no conflicts of interest.

## Supporting information


**Figure S1:** Safety assessment of oral pCO administration in OA rats. (A) Representative H&E staining of liver, kidney and lung tissues. Scale bar = 100 μm. (B) Serum levels of AST, ALT, Cr and BUN.

**Table S1:** Chromatographic gradient from HPLC‐Q‐Orbitrap‐MS.

**Table S2:** Full potential components and targets of pCO identified from the TCMSP database.

**Table S3:** Potential anti‐OA targets of pCO identified from the GeneCards, OMIM and DrugBank databases.

**Table S4:** Topological analysis of the PPI network for pCO targets against OA, including Degree Centrality, Betweenness Centrality and Closeness Centrality.

**Table S5:** D‐C‐T‐P‐D network analysis of potential compounds from pCO in OA, including Degree, Closeness and Betweenness.

## Data Availability

The data that support the findings of this study are available on request from the corresponding author. The data are not publicly available due to privacy or ethical restrictions.
