# Supplementary material for: Wine‐Processed Cornus officinalis Ameliorates Osteoarthritis via Modulating M1/M2 Macrophage Polarization
Source: J Cell Mol Med. 2026 Mar 27;30(7):e71113. doi: 10.1111/jcmm.71113 (PMC13140850; doi:10.1111/jcmm.71113)
Supplement: Supplementary file 1 — Figure S1: Safety assessment of oral pCO administration in OA rats. (A) Representative H&E staining of liver, kidney and lung tissues. Scale bar = 100 μm. (B) Serum levels of AST, ALT, Cr and BUN. [file JCMM-30-e71113-s006.docx]

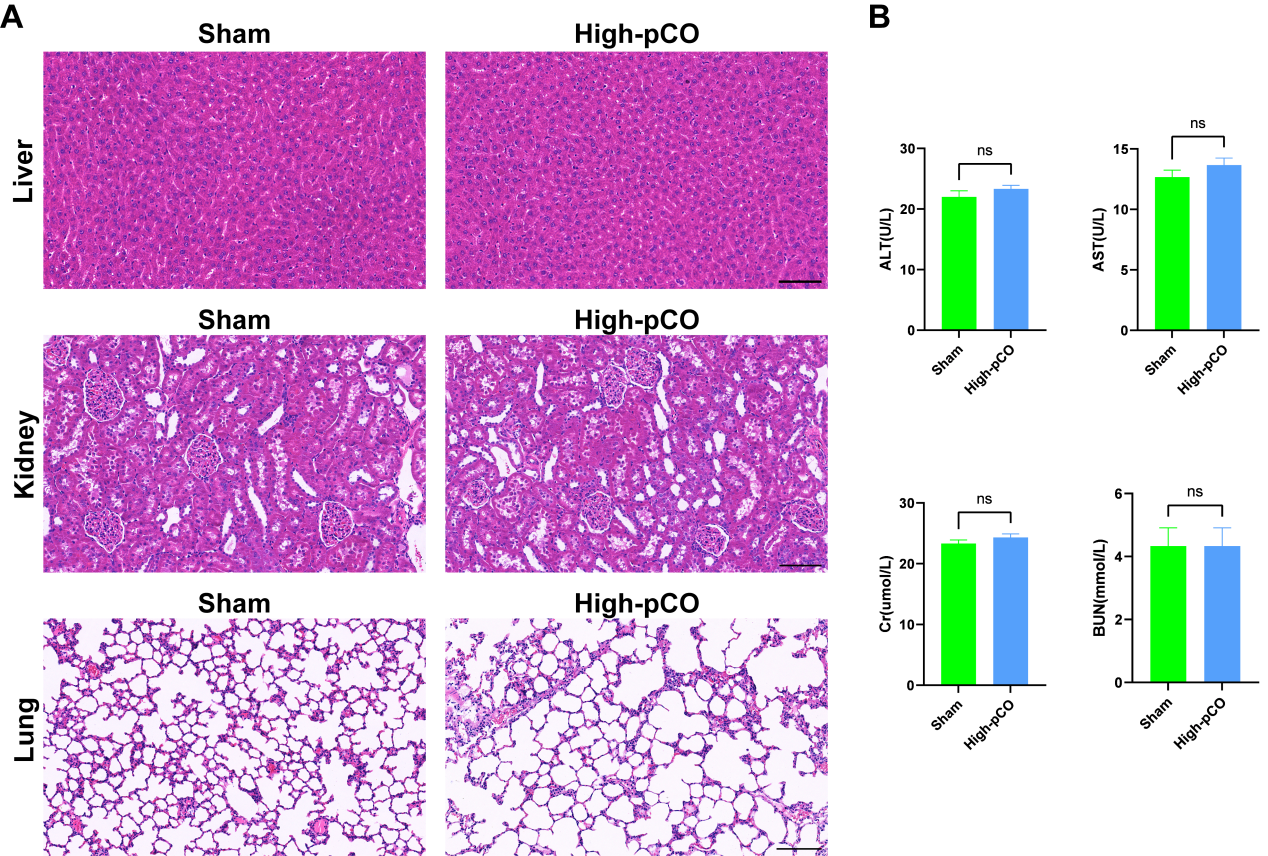


Figure S1. Safety assessment of oral pCO administration in OA rats. (A) Representative H&E staining of liver, kidney, and lung tissues. Scale bar = 100 μm. (B) Serum levels of AST, ALT, Cr, and BUN. Data are expressed as mean ± SD (n = 6). ns P > 0.05 vs sham group.
