# Supplementary material for: Wine‐Processed Cornus officinalis Ameliorates Osteoarthritis via Modulating M1/M2 Macrophage Polarization
Source: J Cell Mol Med. 2026 Mar 27;30(7):e71113. doi: 10.1111/jcmm.71113 (PMC13140850; doi:10.1111/jcmm.71113)
Supplement: Supplementary file 2 — Table S1: Chromatographic gradient from HPLC‐Q‐Orbitrap‐MS. [file JCMM-30-e71113-s002.docx]

**Table S1. Chromatographic gradient from HPLC-Q-Orbitrap-MS.**

| **Time(min)** | **Water phase ratio(%)** | **Organic phase ratio(%)** |
| --- | --- | --- |
| 1 | 98 | 2 |
| 5 | 80 | 20 |
| 10 | 50 | 50 |
| 15 | 20 | 80 |
| 20 | 5 | 95 |
| 27 | 5 | 95 |
| 28 | 98 | 2 |
| 30 | 98 | 2 |
