# Supplementary material for: Wine‐Processed Cornus officinalis Ameliorates Osteoarthritis via Modulating M1/M2 Macrophage Polarization
Source: J Cell Mol Med. 2026 Mar 27;30(7):e71113. doi: 10.1111/jcmm.71113 (PMC13140850; doi:10.1111/jcmm.71113)
Supplement: Supplementary file 3 — Table S2: Full potential components and targets of pCO identified from the TCMSP database. [file JCMM-30-e71113-s001.docx]

**Table S2. Full potential components and targets of** **pCO identified from the TCMSP database.**

| **Database** | **Mol ID** | **Ingredient name** | **Target name** | **Gene name** | **Uniprot ID** |
| --- | --- | --- | --- | --- | --- |
| TCMSP | MOL001494 | Mandenol | Prostaglandin G/H synthase 1 | PTGS1 | P23219 |
| TCMSP | MOL001494 | Mandenol | Prostaglandin G/H synthase 2 | PTGS2 | P35354 |
| TCMSP | MOL001494 | Mandenol | Nuclear receptor coactivator 2 | NCOA2 | Q15596 |
| TCMSP | MOL001495 | Ethyl linolenate | Prostaglandin G/H synthase 1 | PTGS1 | P23219 |
| TCMSP | MOL001495 | Ethyl linolenate | Nuclear receptor coactivator 2 | NCOA2 | Q15596 |
| TCMSP | MOL001771 | poriferast-5-en-3beta-ol | Progesterone receptor | PGR | P06401 |
| TCMSP | MOL001771 | poriferast-5-en-3beta-ol | Nuclear receptor coactivator 2 | NCOA2 | Q15596 |
| TCMSP | MOL002879 | Diop | Sodium channel protein type 5 subunit alpha | SCN5A | Q14524 |
| TCMSP | MOL002879 | Diop | Beta-2 adrenergic receptor | ADRB2 | P07550 |
| TCMSP | MOL002879 | Diop | Muscarinic acetylcholine receptor M3 | CHRM3 | P20309 |
| TCMSP | MOL002883 | Ethyl oleate (NF) | Nuclear receptor coactivator 2 | NCOA2 | Q15596 |
| TCMSP | MOL003137 | Leucanthoside | Proto-oncogene serine/threonine-protein kinase Pim-1 | PIM1 | P11309 |
| TCMSP | MOL000358 | Beta-sitosterol | Progesterone receptor | PGR | P06401 |
| TCMSP | MOL000358 | Beta-sitosterol | Nuclear receptor coactivator 2 | NCOA2 | Q15596 |
| TCMSP | MOL000358 | Beta-sitosterol | Prostaglandin G/H synthase 1 | PTGS1 | P23219 |
| TCMSP | MOL000358 | Beta-sitosterol | Prostaglandin G/H synthase 2 | PTGS2 | P35354 |
| TCMSP | MOL000358 | Beta-sitosterol | Phosphatidylinositol-4,5-bisphosphate 3-kinase catalytic subunit, gamma isoform | PIK3CG | P48736 |
| TCMSP | MOL000358 | Beta-sitosterol | Potassium voltage-gated channel subfamily H member 2 | KCNH2 | Q12809 |
| TCMSP | MOL000358 | Beta-sitosterol | Dopamine D1 receptor | DRD1 | P21728 |
| TCMSP | MOL000358 | Beta-sitosterol | Muscarinic acetylcholine receptor M3 | CHRM3 | P20309 |
| TCMSP | MOL000358 | Beta-sitosterol | Muscarinic acetylcholine receptor M1 | CHRM1 | P11229 |
| TCMSP | MOL000358 | Beta-sitosterol | Sodium channel protein type 5 subunit alpha | SCN5A | Q14524 |
| TCMSP | MOL000358 | Beta-sitosterol | Gamma-aminobutyric-acid receptor alpha-2 subunit | Gabra2 | P47869 |
| TCMSP | MOL000358 | Beta-sitosterol | Muscarinic acetylcholine receptor M4 | CHRM4 | P08173 |
| TCMSP | MOL000358 | Beta-sitosterol | CGMP-inhibited 3',5'-cyclic phosphodiesterase A | PDE3A | Q14432 |
| TCMSP | MOL000358 | Beta-sitosterol | 5-hydroxytryptamine 2A receptor | HTR2A | P28223 |
| TCMSP | MOL000358 | Beta-sitosterol | Gamma-aminobutyric-acid receptor alpha-5 subunit | GABRA5 | P31644 |
| TCMSP | MOL000358 | Beta-sitosterol | Alpha-1A adrenergic receptor | ADRA1A | P35348 |
| TCMSP | MOL000358 | Beta-sitosterol | Gamma-aminobutyric-acid receptor alpha-3 subunit | GABRA3 | P34903 |
| TCMSP | MOL000358 | Beta-sitosterol | Muscarinic acetylcholine receptor M2 | CHRM2 | P08172 |
| TCMSP | MOL000358 | Beta-sitosterol | Alpha-1B adrenergic receptor | ADRA1B | P35368 |
| TCMSP | MOL000358 | Beta-sitosterol | Beta-2 adrenergic receptor | ADRB2 | P07550 |
| TCMSP | MOL000358 | Beta-sitosterol | Neuronal acetylcholine receptor subunit alpha-2 | CHRNA2 | Q15822 |
| TCMSP | MOL000358 | Beta-sitosterol | Sodium-dependent serotonin transporter | SLC6A4 | P31645 |
| TCMSP | MOL000358 | Beta-sitosterol | Mu-type opioid receptor | OPRM1 | P35372 |
| TCMSP | MOL000358 | Beta-sitosterol | Gamma-aminobutyric acid receptor subunit alpha-1 | GABRA1 | P14867 |
| TCMSP | MOL000358 | Beta-sitosterol | Neuronal acetylcholine receptor protein, alpha-7 chain | CHRNA7 | P36544 |
| TCMSP | MOL000358 | Beta-sitosterol | Apoptosis regulator Bcl-2 | BCL2 | P10415 |
| TCMSP | MOL000358 | Beta-sitosterol | Apoptosis regulator BAX | BAX | Q07812 |
| TCMSP | MOL000358 | Beta-sitosterol | Caspase-9 | CASP9 | P55211 |
| TCMSP | MOL000358 | Beta-sitosterol | Transcription factor AP-1 | JUN | P05412 |
| TCMSP | MOL000358 | Beta-sitosterol | Caspase-3 | CASP3 | P42574 |
| TCMSP | MOL000358 | Beta-sitosterol | Caspase-8 | CASP8 | Q14790 |
| TCMSP | MOL000358 | Beta-sitosterol | Protein kinase C alpha type | PRKCA | P17252 |
| TCMSP | MOL000358 | Beta-sitosterol | Transforming growth factor beta-1 | TGFB1 | P01137 |
| TCMSP | MOL000358 | Beta-sitosterol | Serum paraoxonase/arylesterase 1 | PON1 | P27169 |
| TCMSP | MOL000358 | Beta-sitosterol | Microtubule-associated protein 2 | MAP2 | P11137 |
| TCMSP | MOL000359 | Sitosterol | Progesterone receptor | PGR | P06401 |
| TCMSP | MOL000359 | Sitosterol | Nuclear receptor coactivator 2 | NCOA2 | Q15596 |
| TCMSP | MOL000359 | Sitosterol | Mineralocorticoid receptor | NR3C2 | P08235 |
| TCMSP | MOL000449 | Stigmasterol | Progesterone receptor | PGR | P06401 |
| TCMSP | MOL000449 | Stigmasterol | Mineralocorticoid receptor | NR3C2 | P08235 |
| TCMSP | MOL000449 | Stigmasterol | Nuclear receptor coactivator 2 | NCOA2 | Q15596 |
| TCMSP | MOL000449 | Stigmasterol | Alcohol dehydrogenase 1C | ADH1C | P00326 |
| TCMSP | MOL000449 | Stigmasterol | Ig gamma-1 chain C region | IGHG1 | P01857 |
| TCMSP | MOL000449 | Stigmasterol | Retinoic acid receptor RXR-alpha | RXRA | P19793 |
| TCMSP | MOL000449 | Stigmasterol | Nuclear receptor coactivator 1 | NCOA1 | Q15788 |
| TCMSP | MOL000449 | Stigmasterol | Prostaglandin G/H synthase 1 | PTGS1 | P23219 |
| TCMSP | MOL000449 | Stigmasterol | Prostaglandin G/H synthase 2 | PTGS2 | P35354 |
| TCMSP | MOL000449 | Stigmasterol | Alpha-2A adrenergic receptor | ADRA2A | P08913 |
| TCMSP | MOL000449 | Stigmasterol | Sodium-dependent noradrenaline transporter | SLC6A2 | P23975 |
| TCMSP | MOL000449 | Stigmasterol | Sodium-dependent dopamine transporter | SLC6A3 | Q01959 |
| TCMSP | MOL000449 | Stigmasterol | Beta-2 adrenergic receptor | ADRB2 | P07550 |
| TCMSP | MOL000449 | Stigmasterol | Aldose reductase | AKR1B1 | P15121 |
| TCMSP | MOL000449 | Stigmasterol | Urokinase-type plasminogen activator | PLAU | P00749 |
| TCMSP | MOL000449 | Stigmasterol | Leukotriene A-4 hydrolase | LTA4H | P09960 |
| TCMSP | MOL000449 | Stigmasterol | Amine oxidase [flavin-containing] B | MAOB | P27338 |
| TCMSP | MOL000449 | Stigmasterol | Amine oxidase [flavin-containing] A | MAOA | P21397 |
| TCMSP | MOL000449 | Stigmasterol | mRNA of PKA Catalytic Subunit C-alpha | PRKACA | P17612 |
| TCMSP | MOL000449 | Stigmasterol | Chymotrypsinogen B | CTRB1 | P17538 |
| TCMSP | MOL000449 | Stigmasterol | Muscarinic acetylcholine receptor M3 | CHRM3 | P20309 |
| TCMSP | MOL000449 | Stigmasterol | Muscarinic acetylcholine receptor M1 | CHRM1 | P11229 |
| TCMSP | MOL000449 | Stigmasterol | Beta-1 adrenergic receptor | ADRB1 | P08588 |
| TCMSP | MOL000449 | Stigmasterol | Sodium channel protein type 5 subunit alpha | SCN5A | Q14524 |
| TCMSP | MOL000449 | Stigmasterol | 5-hydroxytryptamine 2A receptor | HTR2A | P28223 |
| TCMSP | MOL000449 | Stigmasterol | Alpha-1A adrenergic receptor | ADRA1A | P35348 |
| TCMSP | MOL000449 | Stigmasterol | Gamma-aminobutyric-acid receptor alpha-3 subunit | GABRA3 | P34903 |
| TCMSP | MOL000449 | Stigmasterol | Muscarinic acetylcholine receptor M2 | CHRM2 | P08172 |
| TCMSP | MOL000449 | Stigmasterol | Alpha-1B adrenergic receptor | ADRA1B | P35368 |
| TCMSP | MOL000449 | Stigmasterol | Gamma-aminobutyric acid receptor subunit alpha-1 | GABRA1 | P14867 |
| TCMSP | MOL000449 | Stigmasterol | Neuronal acetylcholine receptor protein, alpha-7 chain | CHRNA7 | P36544 |
| TCMSP | MOL005481 | 2,6,10,14,18-pentamethylicosa-2,6,10,14,18-pentaene | Prostaglandin G/H synthase 2 | PTGS2 | P35354 |
| TCMSP | MOL005503 | Cornudentanone | Thrombin | F2R | P25116 |
| TCMSP | MOL005503 | Cornudentanone | Prostaglandin G/H synthase 2 | PTGS2 | P35354 |
| TCMSP | MOL005503 | Cornudentanone | Nuclear receptor coactivator 2 | NCOA2 | Q15596 |
| TCMSP | MOL005530 | Hydroxygenkwanin | Nitric oxide synthase, inducible | NOS2 | P35228 |
| TCMSP | MOL005530 | Hydroxygenkwanin | Prostaglandin G/H synthase 1 | PTGS1 | P23219 |
| TCMSP | MOL005530 | Hydroxygenkwanin | Prostaglandin G/H synthase 2 | PTGS2 | P35354 |
| TCMSP | MOL005530 | Hydroxygenkwanin | Dipeptidyl peptidase IV | DPP4 | P27487 |
| TCMSP | MOL005530 | Hydroxygenkwanin | Heat shock protein HSP 90 | HSP90AB1 | P08238 |
| TCMSP | MOL005530 | Hydroxygenkwanin | Trypsin-1 | PRSS1 | P07477 |
| TCMSP | MOL005530 | Hydroxygenkwanin | Nuclear receptor coactivator 2 | NCOA2 | Q15596 |
| TCMSP | MOL005530 | Hydroxygenkwanin | Phosphatidylinositol-4,5-bisphosphate 3-kinase catalytic subunit, gamma isoform | PIK3CG | P48736 |
| TCMSP | MOL005531 | Telocinobufagin | Mineralocorticoid receptor | NR3C2 | P08235 |
| TCMSP | MOL005531 | Telocinobufagin | Glucocorticoid receptor | NR3C1 | P04150 |
| TCMSP | MOL008457 | Tetrahydroalstonine | Nitric oxide synthase, inducible | NOS2 | P35228 |
| TCMSP | MOL008457 | Tetrahydroalstonine | Prostaglandin G/H synthase 1 | PTGS1 | P23219 |
| TCMSP | MOL008457 | Tetrahydroalstonine | Dopamine D1 receptor | DRD1 | P21728 |
| TCMSP | MOL008457 | Tetrahydroalstonine | Muscarinic acetylcholine receptor M3 | CHRM3 | P20309 |
| TCMSP | MOL008457 | Tetrahydroalstonine | Thrombin | F2R | P25116 |
| TCMSP | MOL008457 | Tetrahydroalstonine | Potassium voltage-gated channel subfamily H member 2 | KCNH2 | Q12809 |
| TCMSP | MOL008457 | Tetrahydroalstonine | Muscarinic acetylcholine receptor M1 | CHRM1 | P11229 |
| TCMSP | MOL008457 | Tetrahydroalstonine | Androgen receptor | AR | P10275 |
| TCMSP | MOL008457 | Tetrahydroalstonine | Sodium channel protein type 5 subunit alpha | SCN5A | Q14524 |
| TCMSP | MOL008457 | Tetrahydroalstonine | Peroxisome proliferator activated receptor gamma | PPARG | P37231 |
| TCMSP | MOL008457 | Tetrahydroalstonine | Coagulation factor Xa | F10 | P00742 |
| TCMSP | MOL008457 | Tetrahydroalstonine | Muscarinic acetylcholine receptor M5 | CHRM5 | P08912 |
| TCMSP | MOL008457 | Tetrahydroalstonine | Prostaglandin G/H synthase 2 | PTGS2 | P35354 |
| TCMSP | MOL008457 | Tetrahydroalstonine | Alpha-2C adrenergic receptor | ADRA2C | P18825 |
| TCMSP | MOL008457 | Tetrahydroalstonine | Muscarinic acetylcholine receptor M4 | CHRM4 | P08173 |
| TCMSP | MOL008457 | Tetrahydroalstonine | Delta-type opioid receptor | OPRD1 | P41143 |
