# Supplementary material for: Wine‐Processed Cornus officinalis Ameliorates Osteoarthritis via Modulating M1/M2 Macrophage Polarization
Source: J Cell Mol Med. 2026 Mar 27;30(7):e71113. doi: 10.1111/jcmm.71113 (PMC13140850; doi:10.1111/jcmm.71113)
Supplement: Supplementary file 4 — Table S3: Potential anti‐OA targets of pCO identified from the GeneCards, OMIM and DrugBank databases. [file JCMM-30-e71113-s005.docx]

**Table S3.** **Potential anti-OA targets of pCO identified from the GeneCards, OMIM and DrugBank databases.**

| **Database** | **Gene name** |
| --- | --- |
| GeneCards Database | ACAN, COL2A1, SMAD3, FRZB, COMP, H19, MEG3, MATN3, GAS5, GDF5, ASPN, HOTAIR, UFC1, IL1B, KCNK15-AS1, MMP13, PACERR, TMSB4X, PCGEM1, KCNJ5, KCTD13, PMS2P2, COL11A2, CCAL1, TNF, IL6, MMP3, TNFRSF11B, TGFB1, ADAMTS5, COL9A1, MMP1, IL1RN, COL9A2, COL9A3, OS4, OS6, ADAMTS4, CXCL8, IL17A, CILP, MIR140, COL11A1, BGLAP, IL1A, SMAD2, SLC26A2, BMP5, MMP9, TIMP1, TGFBR1, RUNX2, PRG4, CRP, IGF1, SOX9, IL10, IL1R1, COL1A1, DDR2, TRPV4, MGP, LRCH1, FBN1, MATN1, TGFBR2, BMP2, UFSP2, TGFB2, CHI3L1, ADAMTS14, MCF2L, ADAM10, DCN, CTSK, WDR35-DT, TGFB3, TRAPPC2, CLEC3B, COL10A1, COL3A1, SMAD6, AEBP1, UCMA, HFE, VDR, ANKH, MIR146A, LOC109461476, ADIPOQ, LEP, PTPN22, TLR4, SPP1, ESR1, SOD2-OT1, CD36, MMP2, KIF22, LRP5, CLCN7, XIST, IL18, LINC02605, IL2RA, PHEX, ZFAS1, MIR155, EFEMP2, CERNA3, COL5A2, PVT1, LINC01672, IL4, PTHLH, HLA-B, HMGB1, XYLT1, ADAM12, NOS2, BMP7, MALAT1, FTO, FMOD, OFD1, HLA-DRB1, COL1A2, TNFRSF1A, STAT4, COL5A1, IL13, ACP5, CALCA, IBSP, BDNF-AS, MEFV, CSGALNACT1, BMP6, SOST, IL1RAPL2, RETN, CD247, CENPP, CXCL12, DANCR, TNXB, NAMPT, CCL5, TIMP2, IL15, DDRGK1, TP53, ENPP1, COG5, NLRP3, SMAD4, TLR2, CCL3, SIRT1, HIF1A, CASR, MYLK, NGF, MIR98, PLAU, IRF5, MYH11, FGFR3, MAPK1, MIR150, IL2RB, MMP8, ACTA2, MIR132, MIR373, CCN2, CANT1, FAS, GNL3, SLC2A10, FGF2, SNORD15A, LOXL3, SERPINE1, IL2, HNF1A-AS1, DKK1, CTSB, TGFA, MIR34A, IL17F, TNFRSF1B, IHH, ACE, SUPT3H, KRT86, UQCC1, LTBP1, VCAM1, PTH, SOD1, TMX2-CTNND1, FN1, OTULIN, ADAMTSL1, DDH2, RELN, ESR2, COL6A4P1, COL27A1, KLF3-AS1, JUN, SOX5, TNFRSF11A, BTNL2, MAPK8, ENG, IGFBP3, CCL15-CCL14, UFL1, OSM, LOC100130744, ADAMTS3, TYR, GHR, SERPINA1, IL18R1, MIR211, SOD2, CTNNB1, CRH, NFKB1, MIR320A, KL, FBN2, PSMC3, HTRA1, DIO2, OPTC, TNC, PTPN2, SYK, HLA-DQB1, TENT5A, CXCL1, LRP6, DOT1L, HGD, HOTTIP, IL7, CALM1, PLAUR, COLGALT2, NFKBIA, ATP7B, CCN4, SNHG5, CCR6, OPRM1, AGER, MIR641, ROCR, HGF, MAPK14, CXCR4, LAMA5, PTGES, MIR4435-2HG, GLIS3, F2RL1, ALDH1A2, IL4R, SAA1, TLR1, SLC22A4, S100A8, MTHFR, IL11, CASP3, TGM2, CDC5L, MIR145, AP3D1, BMP4, NEAT1, THBS1, POMC, COX5A, ANKRD55, MIR33B, ADAM17, CSF1, MIR335, SERPINA3, CCN6, MIR26A1, ELANE, S100A9, TYRP1, CD244, CIITA, NFKBIL1, RHOB, HBP1, TUG1, PRELP, GLT8D1, MIR146B, FASLG, IL16, MMP14, NME8, CASC2, FOS, FGFR1, CASP1, MIR221, MIR27A, MIR93, NFE2L2, MIR22HG, CCL18, TIMP3, MIR671, ADAMTS9, LOX, COMT, MIF, FGF18, PTH1R, IL23A, GSR, WWP2, CX3CL1, GNPTAB, ELN, LIF, EPAS1, DMP1, POSTN, FSTL1, PLOD1, TLR3, NOG, LTBP3, SCN9A, HSPA5, TLR8, FOSL1, CCR5, FGF1, PBRM1, GDF5-AS1, IGF2, MIR381, F9, MMP7, CCL4, HPGD, SNHG7, HP, MIR18A, MIR9-1, MIR125A, GNAS, DSPP, SNHG16, S100A12, IL5, B2M, HULC, TLR8-AS1, ERG, MIR149, VIP, BAX, CCL20, TP63, SOCS1, NCOR2, CYP26B1, NOTCH1, ITGAM, ITGB1, NLRP1, IL34, TRIP4, STAT3, PIK3R1, SLC39A8, TLR9, MIR26B, CTSD, CPT2, NHERF1, GPR143, NR2E3, CHRNG, IPO8, COG4, MYO18B, SUPT20H, SNHG29, SNHG28, UCA1, FBXL19-AS1, LURAP1L-AS1, AP4B1-AS1, TRAF6, HMOX1, LTA, MIR223, ASTN2, FOXP3, HSPD1, IL6R, CRTAC1, PON1, MIR29A, PITX1, PLEC, NCOA3, SHOX, TBX4, TRPV6, TRAPPC1, ITLN1, CCR3, CHADL, IL37, GPR22, CSF2, LEPR, BGN, LEPQTL1, CX3CR1, PGR-AS1, TGFBR3, MEPE, LPAR1, IL1RL2, MIR210, FKBP5, FGF23, MIR224, WNT5A, PTN, F2, IL22, ACVRL1, GBA1, MIR27B, MIR199A1, SYVN1, BMP1, CAT, CDKN1A, P4HB, CHUK, MIR130A, MIR195, PEPD, CXCL10, MRC1, CLU, HDAC9, CCR7, TRAPPC10, XK, LYPLAL1-AS1, MIR22, LINC01411, IFRD1, LMX1B, NACA2, DUS4L, CCND1, MIR29B1, GPT, AKT1, VIM, FOXO3, EZH2, TNFAIP3, LEF1, IL9, SLBP, CXCR3, DNMT3A, CP, MIR1227, IL21, TLN2, CCN1, PLOD2, FGFR2, BDKRB2, OXT, ANXA1, PWAR1, GJA1, MIR16-1, TLR10, SNHG1, VIM2P, CD163, MIA2, ITIH1, KCNQ1OT1, ZC3H11B, TWIST1, CTBP1-DT, GAPDH, MYC, SP1, MCM3AP-AS1, MIR186, IL33, TRAF1, SIRT6, PAPSS2, ENO1, PROCR, IRAK3, FBLN5, CCL7, IL32, FKBP14, MTOR, CASP8, CXCL13, NOX4, OMD, MIR23A, MIR101-1, IL1RAP, IL17RA, IFNB1, MIR29C, JAG1, MIR138-2, CANX, PARP1, EMSLR, GREM1, UBA5, UFM1, CDK5RAP3, UFSP1, TMEM129, ZEB1, SPG7, SREBF2, HDAC4, RBFOX1, CILP2, MMP17, CD40, HAS2, MIR17, MIR663A, MCAM, HLA-DQA2, PARD3B, MIR142, CYTOR, MIR199A2, CHRD, HSP90B1, HHIP, WNT16, DELEC1, YAP1, MIRLET7E, RNU6-1, MIR126, MIR103A1, MIR423, APOA1, CDH2, DNM3OS, MIR199B, LGALS3, MIR214, HAS1, DNMT1, FOXO1, MIR204, MIR33A, MIR15A, GPI, MIR337, IGFBP7, SMURF2, RARRES2, ALPL, MAF, B3GAT3, CD68, TRAPPC2B, MIR885, DYM, SCARB2, PTEN, MIAT, CEMIP, AQP1, CCR2, BSG, TFRC, CMKLR1, WNT3A, SST, SOX6, MIR21, MIR148A, DEAF1, TRIP11, GORAB, ATF2, TFEB, ITGB2, PLAT, IL1R2, HAVCR2, MAZ, MIR4284, HOTAIRM1, IGFBP5, MIR127, TIMP4, CRYAA, ATG7, CCL16, CCL14, SPCS1, RWDD2B, CCR1, TSPO, NPY, NGFR, PIK3CG, MIR9-2, DLX5, MIR451A, PADI4, EDN1, ANP32A, SLPI, NOD2, ITGB5, SELL, IL10RA, CXCR5, MIR320C1, DPP4, MIR497, MIR376B, TPI1, FLNA, GLB1, LY96, TRPS1, EGF, EFEMP1, ADAMTS1, INHBA, LUM, MMP28, MIR181A1, CRTAP, FKBP10, P3H1, STAT1, LGALS1, OLR1, MIR193B, SDC4, MIR543, KLF5, SNHG14, MIR488, DIO3, MIR500B, CREB1, CHKB-CPT1B, PDIA4, GDI1, SEC23A, SEC23B, RAB33B, TRAPPC4, SAR1A, TRAPPC6B, TRAPPC9, GOLPH3, TRAPPC12, TRAPPC3, TRAPPC2L, TRAPPC11, TRAPPC6A, TRAPPC8, TRAPPC5, TRAPPC13, TRAPPC3L, PIEZO1, MET, CYP19A1, PTGER4, MIR675, MMP12, SLC17A5, P4HA1, LMNA, STT3A, FOLR2, THRA, USP33, LRRK2, MELTF-AS1, NUCB2, CXCL9, CYP27B1, ITGB3, HSPG2, SNORD13, MIR4435-2, LINC-ROR, MYD88, UCN, SFRP4, ITGAV, LOC654780, CAST, TACR1, ADAMTS7, P3H2, MSTN, GNAS-AS1, MIR222, INS, MIR196A2, MIR425, MIR20B, MIR125B1, MIR206, MIR30A, XDH, SDC1, P3H2-AS1, TRE-TTC3-1, LSP1P3, SERPINH1, THBS3, MAN2C1, SHBG, CCR4, IL22RA1, ALOX15, JUND, MIR139, MIR106A, MIR1277, YWHAE, DUSP1, PRDX3, S100B, EDC3, MAP2K6, APOE, HLA-A, CHST11, B3GALT6, NLRC5, MEN1, DNAH8, MIR502, CRELD2, SMAP2, CRLF3, KAZALD1, NFIX, SFRP1, IL1RL1, TRL-TAG1-1, CAMK2B, DPEP1, DVL2, CRTC1, XRCC4, MUS81, TNIP2, CSMD1, DNAH10, TMEM167A, SCARNA18, TSBP1-AS1, PRKAR2B, BCAP29, HPBP, IGF1R, ANPEP, TH, PECAM1, VTN, MC1R, CXADR, HDAC1, TCF4, OPRD1, THY1, MIR181C, CRYAB, KDM6B, LCN2, IL18RAP, MKX, MIR30B, MIR181A2, MIR328, MIR144, MIR24-1, CDR1-AS, AIP, HDAC2, USP50, CD14, PRDX5, SMAD5-AS1, TNFRSF6B, RFX3, AGPAT4, GLIS3-AS2, lnc-GLIS3-2, HSALNG0069708, IKBKE, JUNB, MIR133B, GHRL, SAA4, CDKN2B-AS1, YY1, CTSL, TFAP2A, APOB, FAP, STC1, OLIG3, SNHG15, MIR129-2, MIR9-3, MIR1207, CAV1, MIR216A, CXCR2, MAP2K4, PSTPIP1, PAPPA2, CHI3L2, RMRP, MIR365A, ADIPOR1, PDPN, VWF, RXRB, SLC11A1, MCOLN1, B4GALT7, CHST14, GOLGB1, CEACAM16, TECTB, TNFSF13B, ONECUT2, SLX1A-SULT1A3, SOX4, MIR31, CCL11, TREH, HLA-DQA1, ADCYAP1, DKK3, LRRC15, AKNA, XCL2, PART1, MIR200B, LINC00707, SNORD43, ADRB2, OCRL, TAGLN, PREP, EXT1, GHRHR, PTPRC, FGF7, TRIB3, APLN, MIR483, MIR7-3HG, BMPR1A, MIR582, MIR92A1, GLA, NPC1, ADM2, ANXA5, TNFSF14, CALCR, NR1I3, EMSY, PNOC, DNMT3B, PTK2B, ACTG1, ESRRB, HDAC7, NR1D1, TYMP, NTN1, ACSL4, CALM2, CRLF1, FOXM1, SERPINB2, METTL3, MFGE8, EIF4G2, GPX3, FFAR4, UNC5B, SPON1, FBXO21, MIR141, MIR107, MIR200A, MIR24-2, MIR375, MIR590, MIR128-1, FOXD2-AS1, MIR361, MIR1202, MIR4654, HIVEP2-DT, MIR1246, ADAMTS8, MIR455, SNORD19, PWAR4, EIF6, RPGRIP1L, CHD9, IRX3, KIF12, FTO-IT1, RN7SKP15, RPS20P10, piR-39858-354, HSALNG0111532, LOC124903691, H3C3, SPARC, HPSE, IGFBP2, ROR2, TRC-GCA24-1, PGF, CCL21, PLEKHA7, PCMTD1, MAP3K4, POLG, IL24, MIR296, MIR31HG, CTSG, MIR92B, MMP10, PRTN3, ITGA5, SEMA3A, MIR197, MIR485, GRN, BIRC5, GZMA, TCF7L1, MIR130B, MIR29B2, PDCD5, PRL, IGF2R, PPIB, CD40LG, NTRK1, ALPP, ATF6, TPSAB1, MIR454, TMED2, ANGPTL2, ANGPT2, KNG1, IL3, HMGB2, ADIPOR2, LGALS9, CCL8, CLEC4A, CYTL1, MIR634, SERPINC1, ILF3-DT, COL20A1, UGDH, PTX3, MIR125B2, CHAD, LINC00473, MIRLET7A2, MIR377, SNORD95, IKBKB, F8, SLC40A1, ZMPSTE24, GPR101, ZNF687, LOC101448202, CD4, WARS1, TCIRG1, CD86, OSTM1, ADAMTS20, PALM, PALMD, MIR3922, CYCS, AZU1, MYOSLID, TENM3-AS1, GSK3B, CD38, CNR1, IL7R, LOXL2, SPHK1, HBEGF, SERPINB5, SOD3, PCSK6, SP3, SERPINE2, KLF2, TNFSF12, HLA-DPB1, CRADD, RASGRP3, DYNC1I1, PLXNC1, TAF11, ZNF76, BANK1, TSKU, PLPP6, FAM53A, C17orf67, MMP24OS, MTND4P14, CM034959-017, HSALNG0069709, NONHSAG011935.2, lnc-KIF12-2, USP8, ITIH4, CFI, F3, CSF3, BMAL1, BBC3, CCN3, MIR99A, MIR124-1, PAK1, TRA-TGC7-1, SELE, AGTR1, ERAP2, CHST3, NFAT5, CXCL5, NOS1, BAG6, CHRDL2, KAT5, CEACAM4, TLR5, SERPINA5, NLRP12, WNT9A, TPR, CACNA1G, ATF3, MIR6786, LNCRNA-ATB, HRH4, CXCL11, RPA3, IL36A, LOC111365141, SOCS3, DSP, MMP16, CCL22, HAS2-AS1, GRIN2B, ASAH1, PRKN, PARK7, PSAP, CFL2, LAMP2, IGFBP1, NAGLU, AIM2, SLC26A4, NIN, DNAI2, DDHD1, PACSIN3, ROGDI, ELOF1, NT5E, CSF1R, ALOX5AP, AXL, XBP1, MIR30C1, OIP5-AS1, RAB4B-EGLN2, ADA, LRP1, KCNMA1, H1-4, ETS2, MAP1LC3A, ERGIC3, GC, C3, PPARGC1A, DDR1, KLF4, TGIF1, B4GALT1, CTSS, FBXW7, AHSG, CD47, TRAF2, CST3, GAS6, GPD1L, HMGA2, HYAL2, CABIN1, LGALS8, MRC2, CIRBP, IFNL1, FNDC5, METRNL, MIR338, HCG18, MIR374A, TRD-GTC9-1, OLAH, MSH6, HMGCR, KCNJ11, MUTYH, ORAI1, GLRX, UTS2, EPYC, KLF9, COL6A4P2, CNR2, MIR346, SILC1, GPR33, FAH, EEIG1, CAPN2, ITGA4, PRDM5, SPMIP6, POR, PTGER2, ADM, NRCAM, SIK3, LRG1, NCALD, MIR16-2, GRASLND, MIR4516, CHIT1, ALK, CDKN2A, NOS3, PKM, KEAP1, MAP3K5, ETS1, ITCH, TRAF3, CYBA, RIPK2, ESRRA, FZD8, OPRK1, SHC1, DUSP5, FABP4, PPBP, STT3B, SERPINA4, IL15RA, PITRM1, TFF3, IL27, CCL13, MIR185, MIR1275, PCAT1, ERVK-18, CARD16, ZFP36, SOCS2, NCOA6, SCYL1, AAGAB, SMG6, ACER3, L3MBTL2, LYPLAL1, TIPIN, WSCD2, ZWILCH, BLTP3A, EHBP1L1, PPP1R3B, TMEM18, APOBEC4, IQCH, RFLNA, STIMATE, IQCH-AS1, SNORD16, LINC02742, ENSG00000254632, LINC01875, ENSG00000260773, RNU6-1151P, ENSG00000286417, HSALNG0020226-451, HSALNG0143222, L13304-005, L13304-025, L13715-004, L13715-018, lnc-IQCH-7, lnc-KIF12-3, piR-50208, AB372731, LOC101929770, lnc-LYPLAL1-11, HSALNG0050314, HSALNG0015906, RRM2B, CCL19, LVRN, CAMK2A, IL12A, CSTA, HOXA1, TGIF2, SOX8, FOXD1, C1QTNF9B, MIR4326, E2F1, GNA11, F2RL3, SMURF1, GALE, HIF3A, KLRB1, FIP1L1, DCSTAMP, NUPR1, ZNF440, MIR498, LINC02154, MIR105-1, MIR665, PRNCR1, ARHGAP29-AS1, LINC02888, ENSG00000231760, SMPD1, ADAMTS15, MT-TN, KIT, PIK3CA, PRKCA, RAC1, TLR7, NR1H3, LONP1, XYLT2, C5AR1, TXNRD1, SLC23A2, NMUR1, FGF21, SCG2, PIEZO2, GSTT1, MIRLET7C, LINC01554, NKILA, LOC124900379, TRU-TCA1-1, RNA18SN1, THPO, PYCARD, ADAM15, CTLA4, HSPA4, LTF, TNFRSF10A, JAM3, IL18BP, TREM1, CCK, CRMA, MIR320E, EMILIN1, PLA2G3, EP300, ILF3, CD79A, FURIN, LOC106627981, LOC126861318, LOC126862586, EGFR, BMPR2, MFN2, BRD4, PTK2, CSPG4, HK2, CSF2RA, CS, KPNA2, PTH2R, SFTPA1, ULK1, SFTPB, SFTPD, MAP3K9, SPRED2, DNTT, SFTPC, BNIP3, CBX4, MFAP4, TET1, IL1F10, SLC25A27, SYNE3, C1QTNF12, FER1L4, TLX1NB, LINC00313, MIR9-2HG, DGCR5, MIR122, MIR449A, MIR193A, MIR23B, MIR137, MIR19B1, MIR505, MIR200CHG, MIR300, MIR940, MROCKI, MIR4487, MIR4498, LOC106728418, OAP, TNFSF13, PAX6, HPD, FAM20C, FMR1, CACNA1F, SLC34A1, TRPV5, SLC34A3, NYX, PCOLCE, MAP7, SAA2, YIPF3, ZNF469, KCNJ18, CEBPB, XIAP, APP, KDM1A, ADAMTS2, LAMP3, H3C14, MIR135A1, MIR147B, MIR320B1, MIR320D1, TNFRSF13C |
| OMIM Database | EDM5, FRZB1, GOA1, PLAP1, AGC1, CDMP1, HOA, SRFP3, OS3, CSPG1, SYNS2, OS2, OS1, MSK16, OS5, SEMDBCD, SEDK, BDA1C, SSOAOD, SYM1B, DUPANS |
| Drugbank Database | CYP2C9, PTGS2, PTGS1, CYP1A2, CYP2E1, ABCB1, CYP2D6, CYP2A6, CYP3A4, UGT1A1, UGT1A6, UGT1A9, UGT2B15, SULT1A1, SULT1A3, NAT2, PTGES3, TRPV1, ALB, FAAH, GSTP1, GSTM1, SCN10A, PTGER1, CYP2C19, MPO, MAOA, DBH, GLUL, BCHE, PHB2, PDPK1, ABCC4, CYP2C8, CA2, CA3, ABCB11, CDH11, ABCG2, NEU1, BDNF, GDNF, VEGFA, CCL2, GUSB, HEXB, GALNS, IDS, IDUA, ARSB, TTR, ABCC1, SLC22A6, SLC22A8, SLCO1C1, SLC22A11, UGT2B7, CYP2B6, CYP2C18, SLCO1B1, UGT1A3, UGT2B4, ALOX5, SCN4A, ASIC1, KCNQ2, KCNQ3, PLA2G2A, UGT1A8, RXRA, UGT1A10, SERPINA7, HRH2, SLC22A2, SLC47A1, PPARA, PPARG, IFNG, SLC2A2, SLC2A1, SLC2A4, SLC2A3, RELA, CD44, ICAM1, HMMR, NCAN, VCAN, C1QBP, HAPLN1, HAPLN3, HABP2, LAYN, STAB2, TNFAIP6, IMPG2, HABP4, CEMIP2, ABCC5, LYVE1, SLCO1A2, SLCO2B1, BCL2, THBD, FABP2, CFTR, GP1BA, S100A7, AMACR, CXCR1, SLC22A7, PGD, HSD11B1, AKR1C1, AKR1C2, AKR1C4, pab, UGT1A7, PLA2G1B, CLCNKA, SLC16A7, SLC16A1, PLA2G4A, ATP4A, ABCC3, CYP1A1, CYP1B1, AHR, AR, SERPINA6, NR3C1, SLC22A1, ACHE, AKR1B1, MAPK3, PPARD, PTGDR2, AKR1B10, GLRA1, TNFSF11, TDO2, CYP3A5, CYP3A7 |
