# Supplementary material for: Wine‐Processed Cornus officinalis Ameliorates Osteoarthritis via Modulating M1/M2 Macrophage Polarization
Source: J Cell Mol Med. 2026 Mar 27;30(7):e71113. doi: 10.1111/jcmm.71113 (PMC13140850; doi:10.1111/jcmm.71113)
Supplement: Supplementary file 5 — Table S4: Topological analysis of the PPI network for pCO targets against OA, including Degree Centrality, Betweenness Centrality and Closeness Centrality. [file JCMM-30-e71113-s003.docx]

**Table S4. Topological analysis of the PPI network for pCO targets against OA, including Degree Centrality, Betweenness Centrality, and Closeness Centrality.**

| **Name** | **DegreeCentrality** | **BetweennessCentrality** | **ClosenessCentrality** |
| --- | --- | --- | --- |
| ACHE | 6 | 0.00808974 | 0.575 |
| ADRB2 | 6 | 0.00784059 | 0.575 |
| AKR1B1 | 4 | 0.00122969 | 0.54761905 |
| AR | 8 | 0.00112931 | 0.60526316 |
| BAX | 6 | 0 | 0.575 |
| BCL2 | 16 | 0.04653366 | 0.76666667 |
| CASP3 | 16 | 0.05757974 | 0.76666667 |
| CASP8 | 10 | 0.00519167 | 0.63888889 |
| DPP4 | 5 | 0.00418784 | 0.54761905 |
| JUN | 14 | 0.03378614 | 0.71875 |
| MAOA | 9 | 0.02630449 | 0.62162162 |
| NOS2 | 8 | 0.00387837 | 0.60526316 |
| NR3C1 | 12 | 0.03033573 | 0.67647059 |
| OPRD1 | 3 | 0.00049407 | 0.51111111 |
| OPRM1 | 8 | 0.01996361 | 0.58974359 |
| PIK3CG | 5 | 0.00043917 | 0.56097561 |
| PLAU | 7 | 0.00100383 | 0.58974359 |
| PON1 | 3 | 0.00079051 | 0.51111111 |
| PPARG | 20 | 0.16963254 | 0.88461538 |
| PRKCA | 9 | 0.00695259 | 0.62162162 |
| PTGS1 | 10 | 0.01411632 | 0.63888889 |
| PTGS2 | 20 | 0.18015246 | 0.88461538 |
| RXRA | 4 | 0 | 0.54761905 |
| TGFB1 | 13 | 0.04439956 | 0.6969697 |
