# Supplementary material for: Wine‐Processed Cornus officinalis Ameliorates Osteoarthritis via Modulating M1/M2 Macrophage Polarization
Source: J Cell Mol Med. 2026 Mar 27;30(7):e71113. doi: 10.1111/jcmm.71113 (PMC13140850; doi:10.1111/jcmm.71113)
Supplement: Supplementary file 6 — Table S5: D‐C‐T‐P‐D network analysis of potential compounds from pCO in OA, including Degree, Closeness and Betweenness. [file JCMM-30-e71113-s004.docx]

**Table S5. D-C-T-P-D network analysis of potential compounds from pCO in OA, including Degree, Closeness, and Betweenness.**

| **Name** | **Degree** | **Closeness** | **Betweenness** |
| --- | --- | --- | --- |
| pCO7 | 14.00 | 0.51 | 820.06 |
| pCO14 | 11.00 | 0.44 | 501.79 |
| pCO9 | 8.00 | 0.40 | 333.09 |
| pCO12 | 6.00 | 0.39 | 111.02 |
| pCO1 | 3.00 | 0.36 | 15.88 |
| pCO2 | 2.00 | 0.33 | 2.92 |
| pCO4 | 2.00 | 0.33 | 4.71 |
| pCO10 | 2.00 | 0.36 | 10.32 |
| pCO11 | 2.00 | 0.36 | 10.32 |
| pCO13 | 2.00 | 0.33 | 106.00 |
| pCO3 | 1.00 | 0.33 | 0.00 |
| pCO5 | 1.00 | 0.33 | 0.00 |
| pCO6 | 1.00 | 0.33 | 0.00 |
| pCO8 | 1.00 | 0.33 | 0.00 |
